# Early childhood family threat and longitudinal amygdala-mPFC circuit development: Examining cortical thickness and gray matter-white matter contrast

**DOI:** 10.1016/j.dcn.2024.101462

**Published:** 2024-10-16

**Authors:** Sandra Thijssen, Yllza Xerxa, Linn B. Norbom, Maaike Cima, Henning Tiemeier, Christian K. Tamnes, Ryan L. Muetzel

**Affiliations:** a Behavioral Science Institute, Radboud University, Nijmegen, the Netherlands; b Department of Child and Adolescent Psychiatry/Psychology, Erasmus MC University Medical Center Rotterdam, Rotterdam, the Netherlands; c NORMENT, Division of Mental Health and Addiction, Oslo University Hospital & Institute of Clinical Medicine, University of Oslo, Norway; d PROMENTA Research Center, Department of Psychology, University of Oslo, Norway; e Division of Mental Health and Substance Abuse, Diakonhjemmet Hospital, Oslo, Norway; f Department of Social and Behavioral Sciences, T.H. Chan School of Public Health, Harvard University, Boston, MA, United States; g Department of Radiology and Nuclear Medicine, Erasmus MC University Medical Center Rotterdam, Rotterdam, the Netherlands

**Keywords:** Threat, Neighborhood safety, Parenting, Cortical thickness, Gray-white contrast, Amygdala-mPFC circuit

## Abstract

Early threat-associated cortical thinning may be interpreted as accelerated cortical development. However, non-adaptive processes may show similar macrostructural changes. Examining cortical thickness (CT) together with grey/white-matter contrast (GWC), a proxy for intracortical myelination, may enhance the interpretation of CT findings. In this prospective study, we examined associations between early life family-related threat (harsh parenting, family conflict, and neighborhood safety) and CT and GWC development from late childhood to middle adolescence. MRI was acquired from 4200 children (2069 boys) from the Generation R study at ages 8, 10 and 14 years (in total 6114 scans), of whom 1697 children had >1 scans. Linear mixed effect models were used to examine family factor-by-age interactions on amygdala volume, caudal and rostral anterior cingulate (ACC) and medial orbitofrontal cortex (mOFC) CT and GWC. A neighborhood safety-by-age-interaction was found for rostral ACC GWC, suggesting less developmental change in children from unsafe neighborhoods. Moreover, after more stringent correction for motion, family conflict was associated with greater developmental change in CT but less developmental change in GWC. Results suggest that early threat may blunt ACC GWC development. Our results, therefore, do not provide evidence for accelerated threat-associated structural development of the amygdala-mPFC circuit between ages 8–14 years.

## 1 Introduction

In early childhood, parenting and the larger family environment are imperative in shaping child development. Consequently, early adverse family experiences, such as harsh parental care or parental conflict, can have detrimental effects on mental health and well-being across the life span (e.g. Calhoun et al., 2019; Goulter et al., 2020; Oshri et al., 2018; van Eldik et al., 2020). Several theories attempt to explain how such experiences impact well-being long after the experiences have ended. According to the stress or deficit model of adversity, long-term effects of stressful experiences can be explained by chronic activation of the hypothalamic–pituitary–adrenal (HPA) axis, negatively affecting brain development and ultimately leading to mental health problems (McEwen, 1998, McEwen, 2012). An alternative view on the developmental effects of adversity is inspired by evolutionary theories and suggests that effects of adversity are adaptations to the stressful early life environment (Ellis et al., 2009). One of these theories suggests that early stress may result in accelerated neurodevelopment (Callaghan and Tottenham, 2016). Although this acceleration may provide short-term benefits such as earlier independence, on the long-term, accelerated development has been associated with increased risk of psychopathology (Callaghan and Tottenham, 2016; Mendle et al., 2007).

Studies examining the links between early childhood stress and neurodevelopment have mostly focused on functional connectivity of the amygdala-medial prefrontal cortex (mPFC) circuit, a circuit implicated in emotion regulation (e.g. Gee et al., 2013; Herzberg et al., 2021; Miller et al., 2020; Thijssen et al., 2022; Thijssen et al., 2017). The mPFC, including the anterior cingulate cortex (ACC), is implicated in emotional and cognitive functioning (Forbes and Grafman, 2010, Ridderinkhof et al., 2004) and may provide top-down regulation of amygdala reactivity to emotional stimuli (Etkin et al., 2011). As the mPFC shows a protracted developmental trajectory, parents likely play an important role in child emotion regulation while the mPFC is still immature (Tottenham, 2015). However, with maturation of the amygdala-mPFC circuit, children become increasingly capable of emotional self-regulation. Gee et al. (2013) showed that previously institutionalized youth demonstrated mature amygdala-mPFC circuit functioning earlier in development compared to family-reared youth. Evidence for accelerated development of functional connectivity has also been reported in response to normative variations in the family environment (e.g., Thijssen et al., 2017; Thijssen et al., 2020). Similarly, a few structural magnetic resonance imaging (MRI) studies also show evidence of accelerated brain development following early life stressful experiences (Gur et al., 2019, Thijssen et al., 2020, Tyborowska et al., 2018). Critically, however, most of these studies have relied on cross-sectional (single time-point) imaging data and draw indirect inferences about developmental changes from observed age-related interindividual differences.

Since the 1990’s, a large body of work has been conducted aiming to map normative structural brain development. Longitudinal studies show a monotonous decrease of MRI based cortical thickness over childhood and adolescence (Mills et al., 2014, Nguyen et al., 2013, Tamnes et al., 2017, Walhovd et al., 2016, Wierenga et al., 2014). This thinning was originally thought to be the consequence of activity-dependent synaptic pruning, but more recent studies suggest that myelination of cortical and subjacent axons may be most responsible for the cortex to appear thinner with increasing age (Natu et al., 2019; see further discussion in Norbom et al., 2021; Whitaker et al., 2016). As the cortex appears to thin over the course of development, a thinner cortex or accelerated thinning may be interpreted as accelerated development. However, other non-adaptive processes (e.g., neuronal and dendritic shrinkage in response to high levels of cortisol (Magarinos et al., 1997; Uno et al., 1989) may be responsible for similar macrostructural changes, complicating the differentiation between the deficit or the acceleration hypothesis.

Beyond morphometry, a prominent feature of youth neurodevelopment detected by *in vivo* imaging is an increase in cortical brightness or intensity. Variations in cortical intensity can be assessed by T1-weighted (T1w) intensity metrics such as the grey/white-matter contrast (GWC) (Salat et al., 2009). Given that cholesterol in myelin is a major determinant of the brightness of the T1w signal (Koenig, 1991, Koenig et al., 1990), GWC has been suggested as a proxy for intracortical myelination (Jorgensen et al., 2016). Correspondingly, several studies demonstrate a negative relationship between GWC and age throughout youth and support its effectiveness in mapping global and regional maturational patterns (Drakulich et al., 2021, Lewis et al., 2018, Norbom et al., 2019, Norbom et al., 2020). Intracortical myelination is a crucial feature of postnatal brain development, allowing for efficient signal transmission between neurons and structural support (Bartzokis, 2012). Examining the development of cortical thickness and GWC in concert may enhance the neurobiological interpretation of the association between family-related adversity and cortical development. For example, if an exposure is associated with greater developmental change in thickness *and* GWC, this may be interpreted as evidence that the effect of adversity on thickness represents differences in myelination and thus maturation. If adversity is associated with greater change in thickness but not with GWC, this would be evidence against the effect of adversity on change in thickness representing maturation.

It is unlikely that all adverse family experiences affect child development equally. Prior research on the dimensional model of adversity suggests differential effects of adverse experiences related to threat versus deprivation (Colich et al., 2020, McLaughlin et al., 2014, Sumner et al., 2019), with experiences of threat most consistently related to accelerated development of the mPFC (Colich et al., 2020). Another factor important in determining the impact of adverse experiences on child development may be proximity to the child (Bronfenbrenner, 1977). An influential model that describes the child environment according to its proximity to the child is Bronfenbrenner’s ecological model (Bronfenbrenner, 1977). In this model, the microsystem—which is the system the child has direct experience in— and the child’s interactions within the microsystem, called proximal processes, are influenced by the more distal systems, such as relations and interactions between factors on the microsystem (mesosystem), but also neighborhood (exosystem) or policy (macrosystem). Taking into account the dimensional model of adversity and Bronfenbrenner’s ecological model, the current project will examine whether harsh parenting, family conflict, and neighborhood safety, all threat-related family experiences, but differing in proximity to the child, are associated with the structural development of the amygdala-mPFC circuit. Importantly, all of these factors are potentially associated with accelerated neurodevelopment (Beck et al., 2024, Jedd et al., 2015, Petrican et al., 2021, Peverill et al., 2019, Rakesh et al., 2021).

The current study examines whether early life family threat is prospectively associated with structural development of the amygdala-mPFC circuit by examining both cortical thickness and GWC. Regions of interest were based on prior studies suggesting accelerated functional development of the amygdala-mPFC circuit in response to early stress. Results by Gee et al. (2013) suggest developmental differences in amygdala functional connectivity with a region in the mPFC overlapping with the rostral ACC and medial orbitofrontal cortex (OFC). Results by Thijssen et al. (2017) suggest developmental differences of amygdala functional connectivity in a region overlapping with the caudal ACC. Therefore, we examine harsh parenting, family conflict and neighborhood safety main effects and age-interaction effects on amygdala volume, caudal ACC, rostral ACC, and medial OFC cortical thickness and GWC using data from a large longitudinal cohort, the Generation R Study (Kooijman et al., 2016), with MRI assessments at ages 8, 10, and 14 years old. Based on prior research, we hypothesized that harsh parenting, parental conflict and low neighborhood safety would be associated with accelerated cortical thinning across childhood and adolescence, with more proximate factors having larger effects. We expected that cortical thinning is accompanied by an accelerated decrease of GWC, both indicating accelerated rather than aberrant development. Due to previous conflicting findings (Goddings et al., 2014, Herting et al., 2018, Wierenga et al., 2018), we had no hypotheses with regards to development of amygdala volume, but as prior research in the same sample suggests harsh parenting is associated with lower amygdala volume at age 9, we expect harsh parenting to be negatively associated with amygdala volume.

## 2 Methods

### 2.1 Participants

The current project is embedded within the Generation R Study, a prospective developmental cohort investigating growth, development, and health from fetal life onward in Rotterdam, The Netherlands (Kooijman et al., 2016). Pregnant women living in Rotterdam, the Netherlands, with an expected delivery date between April 2002 and January 2006 were invited to participate. In total, 9778 mothers were enrolled, of which 7393 mothers and their children still participated in mid-childhood. Neuroimaging data was collected in a subset of children between age 6 and 9 years. At ages 10 and 14 years, all children were invited to participate. In the first Generation R imaging assessment, 1070 children were scanned. In the second and third assessments 3992 and 3625 children were scanned, respectively.

For the current study, participants with available predictor(s) and one or more good quality structural MRI scan were included (N= 4471). To avoid correlated datasets, only one child of each twin or sibling pair was included. This led to a total dataset of N = 4200 (2069 boys), of which N = 2759 had data on harsh parenting, N = 3561 had data on family conflict, and N = 3335 had data on neighborhood safety. Of the 4200 included participants, 2503 participants had good quality imaging data at 1 timepoint, 1480 at 2 timepoints, and 217 participants had data at all three time points, leading to a total of 6114 imaging data points.

The Generation R study was approved by the Medical Ethics Committee of the Erasmus Medical Centre, Rotterdam. All parents provided informed consent. Analyses for this study were preregistered at: https://osf.io/yw23h. Deviations from the preregistration are described in Supplementary text 1.

### 2.2 Measures

#### 2.2.1 Family environment

*Harsh parenting:* Information on harsh parenting practices was collected when children were 3 years old using questionnaires based on the Parent-Child Conflict Tactics Scale (CTSPC) (Straus et al., 1998). Items on harsh punishment (e.g., spanking) originally included in the CTSPC were removed, as these practices may be considered illegal in the Netherlands and we had no mandate to follow-up on such practices. Additionally, one item that was not age-appropriate (“said you would kick child out of the house”) was removed, resulting in a 6-item scale. For more information on the construction of the harsh parenting scale, see Jansen et al. (2012). Mothers reported the use of various harsh parenting practices in the preceding 2 weeks, using a 6-point frequency scale (from “Never” to “More than four times”). The internal consistency of harsh parenting was low (Cronbach’s α of 0.63 in the total sample, and 0.55 in the study sample), likely reflecting the small number of items in the scale. Several determinants and correlates of harsh parenting (e.g., socioeconomic status, family dysfunction, child behavioral problems) have been identified in the current cohort (Jansen et al., 2012, Mackenbach et al., 2014) and in the current sample (see Table S2) and support the validity of our harsh parenting measure.

*Family conflict:* Family conflict was assessed using the General Functioning subscale of the Family Assessment Device (FAD, Byles et al., 1988), a validated self-report measure of family health and pathology consisting of 12 items on a 4-point Likert scale (“strongly disagree” to “strongly agree”). Half of the items describe healthy functioning, e.g., ‘In times of crisis, we can turn to each other for support’. The other half describe unhealthy functioning, e.g., ‘There are a lot of unpleasant and painful feelings in our family’. We reverse-coded the six positively-worded healthy-functioning items so that a higher total FAD score indicated less well-functioning families. Mothers completed this measure when their child was 6 years of age (Cronbach’s α =.89).

*Neighborhood safety:* Neighborhood safety scores were obtained from the Municipality of Rotterdam. The neighborhood safety index is based on objective measures of safety (such as police and fire department registries), subjective measures of safety (questionnaires in a sample of neighborhood inhabitants), and context information (such as value of homes, percentage of rental homes) acquired about the year 2007, when children were between 1 and 5 years old. For the subjective measures of safety, 150 respondents from each neighborhood (not necessarily related to the Generation R Study) were asked to fill in a questionnaire regarding neighborhood safety. Respondents included Dutch natives, but also individuals from the five largest minority groups in Rotterdam, the Netherlands (people with Turkish, Moroccan, Surinamese, Antillean and Cape Verdean ancestry). Neighborhood safety scores range from 1 to 10. Neighborhoods with a score below 4 are considered unsafe, a score between 4 and 6 is considered problematic, and a score of 7 or higher is considered safe.

#### 2.2.2 MRI acquisition and processing

MRI scanning at the first imaging assessment was performed on a General Electric (GE) Discovery MR 750 3 T scanner and has been extensively described in White et al. (2013). A T1-weighted inversion recovery fast spoiled gradient recalled (IR-FSPGR) sequence with 0.9 mm isotropic voxel resolution was obtained using an 8-channel head coil. For the second and third assessment, participants were scanned using a different, study-dedicated 3 T GE MR750W. A T1-weighted IR-FSPGR sequence with 1 mm isotropic voxel resolution was obtained using an 8-channel head coil (White et al., 2018). Importantly, as scanner and participant age are correlated, developmental trajectories cannot be meaningfully interpreted (any change in brain structure from visit 1 to a subsequent visit could be due to age or due to change in scanner). However, with the exception of possible effects of attrition bias, variation in early childhood experiences should not be associated with scanner. Moreover, we do not expect the confounded effect of age to differ for variation in the family factors (i.e. the scanner effect should be the same for children growing up in more safe vs more threatening environments). Therefore, differences in developmental trajectory related to these experiences can be interpreted.

Cortical reconstruction, parcellation and volumetric segmentation was performed using FreeSurfer 6.0 (http://surfer.nmr.mgh.harvard.edu/). Cortical thickness was calculated as the shortest vertex-wise distance between the white surface (i.e. gray/white boundary) and the pial surface (i.e. gray/cerebrospinal fluid boundary) (Fischl and Dale, 2000). GWC was calculated using the pctsurfcon function in FreeSurfer (http://surfer.nmr.mgh.harvard.edu/fswiki/pctsurfcon). This function uses intensity sampling from the “rawavg.mgz” volume, with white matter sampled 1 mm below-, and grey matter sampled 30 % above the white surface. The vertex-wise percentage difference was then computed as 100 × (white − grey)/[(white + grey)/2] so that lower GWC reflects more similar grey and white matter.

Region-of-interest were selected form the Desikan-Killiany Atlas (Desikan et al., 2006). Data quality assurance consisted of a multistep process including both visual inspection by trained researchers and automated software and is described elsewhere (Muetzel et al., 2019, Steenkamp et al., 2022, Weeland et al., 2021). Only scans of sufficient quality were used for data analysis. For more information on MRI acquisition and processing, see Supplemental Text 2.

#### 2.2.3 Confounding variables

Analyses were corrected for sex, age, gestational age, parental national origin, family income, maternal education, maternal psychopathology, and smoking during pregnancy. Information on sex, gestational age, and date of birth was obtained from midwives and hospital registries. Family national origin, family income, maternal education, maternal psychopathology and smoking during pregnancy were assessed through questionnaires. For more information on the confounding variables, see Supplemental Text 3.

### 2.3 Statistical analyses

The current region-of-interest MRI project examined the interaction between early family threat and age on amygdala-mPFC circuit structure and microstructure using linear mixed effects modelling in R using the lme4 package (Bates et al., 2015). Prior to analyses, missing values for the confounding variables (1–34 %) were imputed, generating 30 imputed datasets using 50 iterations. Moreover, amygdala volumes were residualized for intracranial volume (analyses were performed on both the uncorrected and corrected amygdala volumes). To examine the association between harsh parenting, family conflict and neighborhood safety and child amygdala-mPFC circuit structure and microstructure at each wave separately, partial correlations were performed correcting for age, sex, family national origin, maternal education, family income, maternal psychopathology, prenatal smoking, gestational age at birth.

Before examining whether the family factors are associated with the development of amygdala-mPFC circuit structure and microstructure, first, the developmental trajectory of each outcome was examined by testing linear and quadratic age effects, using the following formulas:

Y_outcome_ = intercept + random (subject) + age + error, and

Y_outcome_ = intercept + random (subject) + age + age^2^ + error

Because of the limited number of data points per participant, random age slopes were not modelled. Best fitting models were selected based on the likelihood ratio test p-values (p <.05) and Akaike Information Criterion (AIC). The selected models were then used to examine the age-by-family factor interaction effect. To assess the effects of the confounding variables on the age-by-family factor interaction effect, the age-by-family factor interaction effect was tested in three models increasing in the number of confounding variables adjusted for. In the first model, age-by-family factors were adjusted for child sex, family national origin, family income, and maternal education:

Y_outcome_ = intercept + random (subject) + age + family factor + age*family factor + sex + national origin + income + maternal education + error, or

Y_outcome_ = intercept + random (subject) + age + family factor + age^2^ + age*family factor + age^2^*family factor + sex + national origin + income + maternal education + error, for linear and quadratic models, respectively

In the second model we additionally adjusted for gestational age, maternal psychopathology, and maternal smoking during pregnancy. The third and fully adjusted model was additionally corrected for the other family factors of interest. For models in which the family factor-by-age interaction effect was not significant, analyses were performed examining the main effect of the family factor:

Y_outcome_ = intercept + random (subject) + age + family factor + sex + national origin + income + maternal education + error, or

Y_outcome_ = intercept + random (subject) + age + family factor + age^2^ + sex + national origin + income + maternal education + error, for linear and quadratic models, respectively. We also explored age-by-sex-by-family factor interaction effects. The afex package (https://rdocumentation.org/packages/afex/versions/0.28–0) was used to compute statistical significance and the mitml package was used to pool the outcomes of the multiply imputed dataset (https://cran.r-project.org/web/packages/mitml). Results were corrected for multiple comparisons using false discovery rate (FDR) within family factor (7 tests, Benjamini and Hochberg, 1995).

#### 2.3.1 Sensitivity and exploratory analyses

Sensitivity analyses were performed using data from the second and third assessment only to assess the robustness of our findings when data from a single scanner was used. We further performed analyses additionally correcting for motion by correcting for the number of surface holes. The number of surface holes is a quality measure provided by FreeSurfer. Surface holes and the associated Euler number have been shown to correlate with manual quality ratings across samples (Rosen et al., 2018). Finally, to assess whether developmental differences for thickness and GWC are independent, significant associations for cortical thickness were additionally adjusted for GWC and vice versa.

To elucidate the association between development of cortical thickness and GWC, the association between change (T2-T1 and T3-T2) in cortical thickness and change in GWC were examined using linear regression analyses correcting for age (at T1 for the T1-T2 change analyses, and at T2 for the T2-T3 change analyses), Δage and sex. Results can be found in Supplemental Text 4.

### 2.4 Non-response analysis

Compared to the original Generation R sample, included participants were more likely to be girls (*p* =.020, φ =.024), Dutch (*p* <.001, φ =.12), and have higher educated mothers (*p* <.001, φ =.15). Included participants were from safer neighborhoods (*p* <.001, Cohens d = −0.19), higher income families (*p* <.001, Cohens d = −0.13), had a mother with a lower psychopathology score (*p* <.001, Cohens d = 0.13), were less likely to have been exposed to prenatal smoking (*p* <.001, φ =.06), and had a higher gestational age (*p* <.001, Cohens d = −0.10). No differences were found for harsh parenting (*p* =.148) or family conflict (*p* =.771). However, these latter variables were available in less participants than the demographic variables and null-results may be accounted for by sample size (n = +/- 5000 for harsh parenting and 6000 for family conflict, while n = +/- 8500 for prenatal smoking and maternal education). Mean differences in family factors between the participants in the first and subsequent imaging assessments can be found in Table S2.

## 3 Results

Demographic characteristics of the sample can be found in Table 1. All predictor variables were significantly correlated. Harsh parenting was associated with more family conflict (r =.15, *p* <.001) and lower neighborhood safety (r = −.08, *p* <.001) and family conflict was negatively associated with neighborhood safety (r = −.16, *p* <.001). Correlations between predictor and confounding variables can be found in Table S3. For correlations between imaging measures, see Table S4.

**Table 1:** Sample characteristics (N=4200).

	N	M(SD)/N(%)
Boys (n(%))	4199	2069 (49.3)
Age t1	901	7.97 (0.99)
Age t2	3013	10.13 (0.59)
Age t3	2200	14.02 (0.62)
Ethnicity	4200	
Dutch (n(%))		2478 (59.0)
European (n(%))		312 (7.4)
Non-European (n(%))		1410 (33.6)
Maternal education	4079	
Primary		261 (6.4)
Secondary		1709 (41.9)
Higher		2109 (50.2)
Family income	3436	7.20 (2.76)
Harsh parenting	2759	2.18 (1.90)
Family conflict	3561	1.53 (0.43)
Neighborhood safety	3335	7.70 (1.60)
Gestational age at birth	4169	39.82 (1.85)
Maternal smoking	3702	
Never (n(%))		2889 (68.8)
Quit when pregnancy known (n(%))		276 (6.6)
Continued (n(%))		537 (12.8)
Maternal psychopathology	2792	0.16 (0.26)

### 3.1 Developmental trajectories

For model selection criteria, see Table S5. Figure S2 shows the developmental trajectories of the ROIs. Except for rostral ACC cortical thickness and GWC, which showed a linear developmental trajectory, all structural brain measures showed evidence of a quadratic developmental trajectory (Table 2). In general, cortical thickness showed a (decelerating) decrease with age, whereas amygdala volume and GWC showed a (decelerating) increase with age. However, a change in scanner took place between assessment 1 and assessment 2. Developmental trajectories should therefore be interpreted with caution and mostly serve interpretation of environmental effects.

**Table 2:** Associations between age and amygdala-mPFC circuit structure and microstructure.

	Volume/cortical thickness		GWC	
	Age β (SE)	Age^2^ β (SE)	Age β (SE)	Age^2^ β (SE)
Amygdala	0.26 (0.01)	−0.17 (0.01)		
Caudal ACC	−0.35 (0.01)	0.14 (0.01)	0.09 (0.01)	−0.17 (0.01)
Rostral ACC	−0.42 (0.01)		0.35 (0.01)	
mOFC	−0.67 (0.01)	0.11 (0.01)	0.41 (0.01)	−0.15 (0.01)

Note. ACC = anterior cingulate cortex; GWC = gray-white contrast; OFC = orbitofrontal cortex

### 3.2 Associations between early family environment and amygdala-mPFC circuit structure

Below we describe the results of the longitudinal analyses. For partial correlations between family threat factors and brain structure per data wave, see Table S6.

#### 3.2.1 Harsh parenting

**Fig. 1:**
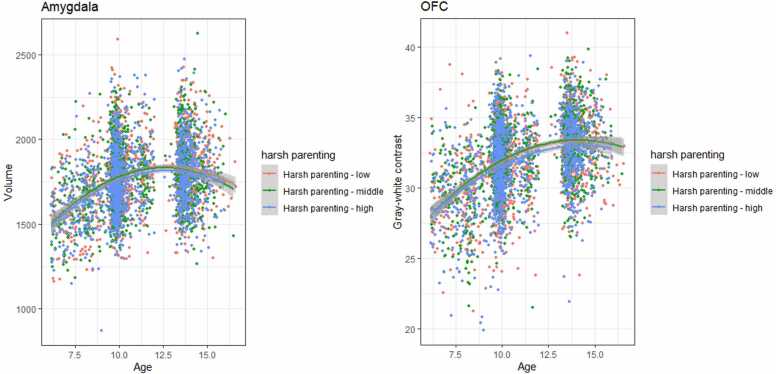
Associations between harsh parenting and amygdala volume and OFC gray-white contrast.

Results for the linear mixed effects models examining the association between harsh parenting and development of amygdala-mPFC circuit structure can be found in Table 3 and Fig. 1. There were no significant interactions between harsh parenting and age(^2^). Significant main effects indicated that harsh parenting was associated with a smaller amygdala volume (uncorrected for TBV, β = −0.05,*p* =.001, *p*_adj_ =.007) and lower OFC GWC (β = −0.04, *p* =.015, *p*_adj_ =.015) across development. When additionally adjusted for gestational age, maternal psychopathology, and maternal smoking during pregnancy, the latter effect was no longer significant after correction for multiple testing (β = −0.03, *p* =.037, *p*_adj_ =.130).

**Table 3:** Linear mixed effect models of harsh parenting.

Structure	Effect	Model 1		Model 2		Model 3	
		β	p	β	p	β	p
Amygdala volume (TBV corr)	Age interaction	0.00	.723	0.00	.723	−0.01	.712
	Main effect	−0.03	.108	−0.03	.065	−0.04	.088
Amygdala volume	Age interaction	0.00	.671	0.00	.695	−0.00	.669
	Main effect	−0.05	.001 (.007)	−0.06	.001 (.007)	−0.06	.004 (.024)
Caudal ACC thickness	Age interaction	0.00	.828	0.00	.834	0.00	.997
	Main effect	−0.00	.863	−0.01	.524	0.01	.807
Caudal ACC GWC	Age interaction	0.02	.243	0.02	.230	0.03	.082
	Main effect	−0.00	.816	−0.00	.976	0.00	.947
Rostral ACC thickness	Age interaction	−0.01	.357	−0.01	.371	−0.01	.471
	Main effect	−0.01	.401	−0.02	.292	−0.02	.251
Rostral ACC GWC	Age interaction	−0.01	.428	−0.01	.447	−0.02	.241
	Main effect	0.00	.956	0.00	.877	−0.00	.858
mOFC thickness	Age interaction	−0.01	.299	−0.01	.304	−0.01	.515
	Main effect	−0.01	.608	−0.01	.599	−0.02	.388
mOFC GWC	Age interaction	0.00	.648	0.01	.640	0.00	.948
	Main effect	−0.04	.015 (.015)	−0.03	.037 (.130)	−0.02	.232

Note: model 1: adjusted for sex, family national origin, family income, and maternal education. Model 2 = model 1 + gestational age, maternal psychopathology, and maternal smoking during pregnancy. Model 3 = model 2 + family conflict, neighborhood safety ACC = anterior cingulate cortex; GWC = gray-white contrast; OFC = medial orbitofrontal cortex

#### 3.2.2 Family conflict

**Fig. 2:**
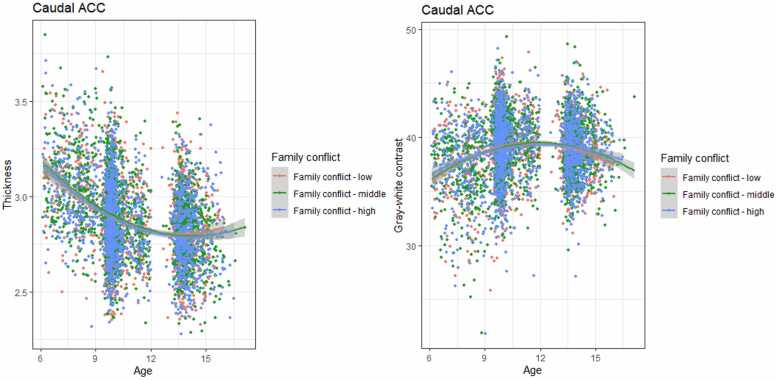
Family conflict-by-age interaction effects on caudal ACC thickness and gray-white contrast.

Results for the linear mixed effects models examining the association between family conflict and development of amygdala-mPFC circuit structure can be found in Table 4. No interaction or main effects were found after correcting for multiple comparisons. Uncorrected conflict-by-age^2^ interaction effects were found for caudal ACC thickness and GWC (β = −0.02, *p* =.018, *p*_adj_ =.063, and β = 0.03 *p* =.010, *p*_adj_ =.063, for thickness and GWC, respectively), and suggest larger developmental change in cortical thickness in high conflict families, whereas low conflict families showed higher rates of developmental change in GWC (Fig. 2).

**Table 4:** Linear mixed effect models of family conflict.

Structure	Effect	Model 1		Model 2		Model 3	
		β	p	β	p	β	p
Amygdala volume (TBV corr)	Age interaction	−0.01	.234	−0.01	.236	−0.02	.170
	Main effect	0.01	.616	0.01	.770	0.03	.257
Amygdala volume	Age interaction	−0.01	.424	−0.01	.434	−0.01	.282
	Main effect	−0.01	.562	−0.01	.541	0.01	.574
Caudal ACC thickness	Age interaction	−0.02	.015 (.053)	−0.02	.018 (.063)	−0.02	.101
Caudal ACC GWC	Age interaction	0.03	.010 (.053)	0.03	.010 (.063)	0.03	.057
Rostral ACC thickness	Age interaction	0.00	.889	0.00	.829	−0.00	.869
	Main effect	0.00	.560	0.01	.679	0.02	.282
Rostral ACC GWC	Age interaction	−0.00	.767	−0.00	.777	−0.00	.939
	Main effect	−0.01	.739	0.00	.733	0.02	.410
mOFC thickness	Age interaction	−0.00	.856	−0.00	.847	−0.00	.859
	Main effect	0.01	.246	0.02	.253	0.02	.338
mOFC GWC	Age interaction	0.01	.455	0.01	.478	−0.00	.917
	Main effect	−0.02	.275	−0.02	.293	−0.01	.535

Note: model 1: adjusted for sex, family national origin, family income, and maternal education. Model 2 = model 1 + gestational age, maternal psychopathology, and maternal smoking during pregnancy. Model 3 = model 2 + harsh parenting, neighborhood safety ACC = anterior cingulate cortex; GWC = gray-white contrast; mOFC = medial orbitofrontal cortex

#### 3.2.3 Neighborhood safety

**Fig. 3:**
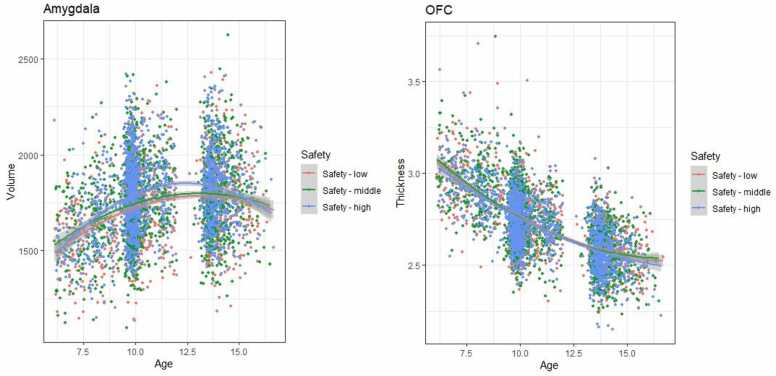
Associations between neighborhood safety and amygdala volume and mOFC cortical thickness.

**Fig. 4:**
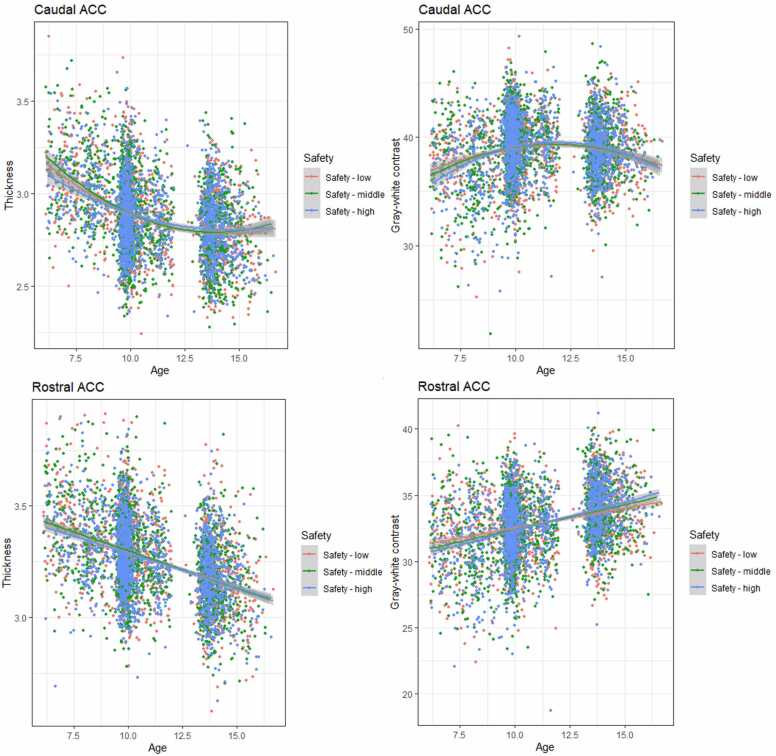
Associations between neighborhood safety and ACC thickness and GWC.

Results for the linear mixed effects models examining the association between neighborhood safety and development of amygdala-mPFC circuit structure can be found in Table 5 and Fig. 3, Fig. 4. Only for rostral ACC GWC a significant safety-by-age interaction effect was found (β = 0.05, *p* <.001), suggesting greater developmental change in children from safer neighborhoods. The fully corrected model of the age-by-safety interaction on caudal ACC thickness was significant, but did not survive correction for multiple testing, β = −0.03, *p* =.011, *p*_adj_ =.077). Like the results for family conflict, interactions indicated greater developmental change in cortical thickness, and lesser developmental change in GWC for children growing up in more dangerous neighborhoods.

**Table 5:** Linear mixed effect models of neighborhood safety.

Structure	Effect	Model 1		Model 2		Model 3	
		β	p	β	p	β	p
Amygdala volume (TBV corr)	Age interaction	−0.01	.468	−0.01	.509	0.01	.379
	Main effect	0.04	.030 (.070)	0.04	.023 (.054)	0.06	.013 (.045)
Amygdala volume	Age interaction	−0.01	.414	0.00	.695	0.01	.441
	Main effect	0.05	.001 (.007)	0.05	.001 (.007)	0.05	.009 (.045)
Caudal ACC thickness	Age interaction	−0.00	.913	−0.00	.860	−0.03	.011 (.077)
	Main effect	−0.00	.817	−0.00	.901	−0.01	.632
Caudal ACC GWC	Age interaction	−0.03	.065	−0.03	.071	−0.01	.561
	Main effect	−0.01	.475	−0.01	.493	−0.01	.512
Rostral ACC thickness	Age interaction	0.01	.079	0.02	.085	0.02	.073
	Main effect	−0.01	.623	−0.01	.676	0.01	.816
Rostral ACC GWC	Age interaction	0.05	<.001 (.001)	0.05	<.001 (.001)	0.04	.035 (.082)
mOFC thickness	Age interaction	−0.01	.270	−0.01	.282	−0.02	.088
	Main effect	−0.03	.018 (.063)	−0.03	.019 (.054)	−0.04	.035 (.082)
mOFC GWC	Age interaction	−0.00	.748	0.01	.589	0.01	.650
	Main effect	−0.01	.485	−0.01	.499	−0.01	.468

Note: model 1: adjusted for sex, family national origin, family income, and maternal education. Model 2 = model 1 + gestational age, maternal psychopathology, and maternal smoking during pregnancy. Model 3 = model 2 + harsh parenting, family conflict ACC = anterior cingulate cortex; GWC = gray-white contrast; mOFC = medial orbitofrontal cortex

Analyses of main effects show that neighborhood safety was associated with higher amygdala volume (uncorrected for TBV β = 0.05, *p* =.001, *p*_adj_ =.007).

### 3.3 Sensitivity analyses

Results for the sensitivity analyses can be found in Tables S7-S13, and Figures S3-S5. Briefly, analyses examining only imaging assessment 2 and 3 data resulted in largely the same results (Table S7-S9, Figure S3-S5). With the exception of the main effect of harsh parenting on mOFC GWC, all significant associations were confirmed using data from assessment 2 and 3 only. The correction for motion resulted in consistently stronger associations, sometimes leading to different interpretations (Tables S10-12). Specifically, the main effect for harsh parenting on medial OFC GWC now survived correction for multiple testing, as did the age-by-family conflict interaction effects on caudal ACC thickness and GWC. For neighborhood safety, the age interaction on caudal ACC GWC was significant but did not survive correction for multiple testing, while the main effects for medial OFC thickness and amygdala volume (TBV corrected) now did survive correction for multiple testing. All associations found for cortical thickness were similar in effect size once corrected for GWC and vice versa (Table S13).

## 4 Discussion

The current study examined whether early family-related threat was associated with structural development of the amygdala-mPFC circuit. Specifically, we examined whether early childhood harsh parenting, family conflict, and neighborhood safety—all factors associated with the family environment but differing in proximity to the child—were prospectively associated with the development of amygdala volume and caudal and rostral ACC and medial OFC cortical thickness and GWC from age 8–14 years old. Results suggest that early threat was not associated with developmental differences in amygdala volume or OFC structure. However, harsh parenting and lower neighborhood safety were associated with smaller amygdala volume across age. For rostral ACC GWC, developmental differences were found related to neighborhood safety. Specifically, lesser developmental change in rostral ACC GWC was found in children growing up in more dangerous neighborhoods.

In line with prior research, partly from the same sample, for harsh parenting and neighborhood safety but not for family conflict, independent associations with amygdala volume were found (Cortes Hidalgo et al., 2022, Saxbe et al., 2018, Xerxa et al., 2021). In prior studies, neighborhood violence and threat have been found associated with both reduced (Saxbe et al., 2018) and increased amygdala volume (Orendain et al., 2023). While Saxbe et al. (2018) examined a relatively small sample of adolescents, Orendain et al. (2023) made use of data from a large population-based cohort, the Adolescent Brain and Cognitive Development (ABCD) Study. In their cross-sectional study, Orendain et al. (2023) related perceived threat (i.e., answer to the question “*My neighborhood is safe from crime*”) to amygdala volume in 9–10 years old children. Our measure of neighborhood safety is based on objective measures of safety (such as police and fire department registries), subjective measures of safety (questionnaires in a sample of neighborhood inhabitants), and context information (such as value of homes, percentage of rental homes) acquired when children were between 1 and 5 years old. Differences in age of exposure as well as type of measurement (self-report vs external data) and cultural differences (USA vs the Netherlands) could explain the different direction of the association. Future studies exploring the effect of timing of exposure to neighborhood safety across cultures may provide more insight in the association between neighborhood safety and amygdala volume.

The negative association between amygdala volume and harsh parenting has previously been reported in the same sample at age 10 years old (Cortes Hidalgo et al., 2022), but was also found in different samples (Suffren et al., 2022). Similar to Whittle et al. (2022), we do not find developmental differences in amygdala volume in response to harsh parental practices. Thus, although amygdala volume seems sensitive to exposure to threat on both proximal and more distal levels, it does not show evidence of threat-related developmental differences from the middle-childhood to early-adolescence period.

Similar to amygdala volume, no evidence was found for threat-related developmental differences in medial OFC structure. Our results do suggest that exposure to more distal early family-related threat is associated with development of the ACC. For neighborhood safety, an age-interaction effect suggested greater developmental change in rostral ACC GWC in children growing up in safer environments. Our results, therefore, do not provide evidence for accelerated development of the amygdala-mPFC circuit in this age-range, but instead could be interpreted as decelerated or blunted development in relation to neighborhood unsafety. Interestingly, using data from the ABCD Study, a recent study suggests neighborhood safety is associated with increased brain age in children aged 9–13 years old (Beck et al., 2024). In this study, brain age was computed from several T1w metrics, including cortical thickness and GWC. Although growing up in an unsafe neighborhood may be associated with older looking brains on average, our results suggest that for specific metrics results may differ and depend on age.

Indeed, for both family conflict and neighborhood safety, we found uncorrected age-interactions that potentially corroborate the suggestion that early threat may be differentially associated with different T1w metrics. Importantly, these interaction effects did survive correction for multiple testing after more stringent adjustment for motion, and suggest greater developmental change in caudal and/or rostral ACC thickness in individuals from high conflict or low safety families, whereas for GWC, higher family conflict or lower neighborhood safety was associated with less developmental change. These findings seem to partly support accelerated development of cortical thickness in response to early threatening experiences (Thijssen et al., 2020), whereas for development of GWC a consistent decelerated or blunted pattern is found. Speculatively, these findings may suggest that accelerated cortical thinning in response to early threat does not represent accelerated cortical development, or could suggest that in different environments, different developmental processes are favored (i.e., pruning in threatening environments, myelination in safer environments). However, not much is known about the concurrent developmental patterns of cortical thickness and GWC (Norbom et al., 2021). Although a recent study suggests that the maturation of cortical thickness is indeed associated to the development of white matter connections (Liang et al., 2024), a study in 127 adults aged 18–81 years suggests that—despite showing similar correlations with age—the spatial distributions of the age trajectories of cortical thickness and GWC are not correlated during aging (Parent et al., 2023). In exploratory analyses examining the correlation between change in thickness and GWC, we found a small negative association between change in thickness and GWC from age 10–14-years old, suggesting that a larger decrease in thickness is associated with a smaller decrease or larger increase in GWC. For rostral ACC, the correlation in change from 8 to 10 and from 10-to 14-years old was small but positive. This seems to imply that a larger decrease in thickness, goes hand in hand in with a smaller increase in GWC, and therefore corresponds to the nature of our interaction effect. Importantly, in the preregistered analyses, only the age-by-neighborhood safety interaction effect on rostral ACC GWC survived correction for multiple testing, and these results therefore should be treated with caution. However, sensitivity analyses correcting for motion suggest other age-interaction effects survive correction for multiple testing also, increasing our confidence in these findings.

Although we hypothesized larger developmental effects for family factors more proximate to the child, only main effects but no age-interactions were reported for our proximate factor of harsh parenting. Surprisingly, for neighborhood safety and family conflict, (uncorrected) interactions with age were found. Potentially, these differential effects can be explained by the difference in saliency of these factors at different developmental stages (Hyde et al., 2022). Speculatively, as parenting is the most salient environmental factor in early childhood, effects of parenting on structure of the amygdala-mPFC circuit occur earlier in childhood and remain stable thereafter. More distal factors become more salient later in development, and consequently, their effect may only start to occur then. Importantly, however, the interaction effects were not independent of the other family factors (i.e. the neighborhood safety-by-age interaction did not survive adjustment for harsh parenting and family conflict), suggesting specificity of the different measures of threat is limited and effects may be driven by the common factor of threat. The only association independent of the other family factors was the main effect of neighborhood safety on amygdala volume, suggesting neighborhood safety may be an especially salient exposure and/or the amygdala may be especially susceptible to different levels of threat. Interestingly, neighborhood factors only recently have become the focus of neuroimaging studies (e.g. Demidenko et al., 2021; Gard et al., 2022; Hackman et al., 2021; Rakesh et al., 2021). Our results suggest such factors provide a promising avenue for further research.

Although prior cross-sectional research indicates a general decrease of GWC from childhood to adolescence (Norbom et al., 2019, Drakulich et al., 2021), results by Norbom et al. (2019) suggest that GWC of the medial OFC and ACC specifically increases in childhood and adolescence. Our longitudinal data provide support for this deviating developmental pattern of ACC and medial OFC GWC. It is important, however, to note once more that a scanner change occurred during our study between assessment 1 and 2. However, as the increase in GWC was also found between assessment 2 and 3, the positive association between age and GWC seems not accounted for by the scanner change. To extend our knowledge on GWC development, future studies should examine developmental trajectories of GWC using longitudinal data and explore associations with other structural modalities.

Despite considerable strengths, such as the large sample size and the use of multiple T1w metrics, several limitations should be acknowledged. First, after the first wave of data collection, a change in scanner occurred, limiting our ability to interpret developmental trajectories. However, unlike age, exposure and scanner change should not be correlated, nor should the confounded effect of age to differ for variation in the family factors. Therefore, interaction effects with age can be interpreted. Moreover, similar results were found for sensitivity analyses using data from assessment 2 and 3 only, providing evidence our findings cannot be accounted for by the scanner change. Second, due to the large sample size, small effects can become significant. At the population level, however, even small effect sizes, such as those observed here, are potentially meaningful (Dick et al., 2021). Finally, the current sample is a population-based cohort recruited from the Netherlands and includes relatively few participants exposed to high levels of threat. Our findings, therefore, may be not be generalizable to children growing up in more threatening environments.

In conclusion, the present study suggests that early life exposure to family threat is associated with smaller amygdala volume across development. Moreover, less neighborhood safety was associated with less developmental change in rostral ACC GWC. No associations between early family-related threat and medial OFC development were found. Our findings, therefore, provide no consistent evidence for accelerated development of structure of the amygdala-mPFC circuit in response to early family-related threat.

## Funding

The general design of Generation R Study is made possible by financial support from the Erasmus Medical Center, Rotterdam, the Erasmus University Rotterdam, ZonMw, the Netherlands Organisation for Scientific Research (10.13039/501100003246NWO), and the Ministry of Health, Welfare and Sport. The work of HT and RM is supported by the 10.13039/501100000780European Union’s Horizon 2020 Research and Innovation Programme (FAMILY, grant agreement No 101057529). Supercomputing resources for image analysis were provided by the Dutch Organization for Scientific Research (Surf, NWO, 2021.042 to RLM). The Generation R Study is conducted by the Erasmus Medical Center in close collaboration with the School of Law and Faculty of Social Sciences of the Erasmus University Rotterdam, the Municipal Health Service Rotterdam area, Rotterdam, the Rotterdam Homecare Foundation, Rotterdam and the Stichting Trombosedienst & Artsenlaboratorium Rijnmond (STAR-MDC), Rotterdam. We gratefully acknowledge the contribution of children and parents, general practitioners, hospitals, midwives and pharmacies in Rotterdam. C.K.T. was supported by the Research Council of Norway (#223273, #288083, #323951) and the South-Eastern Norway Regional Health Authority #2021070, #2023012, #500189). S.T. was supported by the Dutch Organization for Scientific Research (406.XS.04.166)

## CRediT authorship contribution statement

**Ryan L. Muetzel:** Writing – review & editing, Project administration, Methodology. **Christian K. Tamnes:** Writing – review & editing, Methodology. **Henning Tiemeier:** Writing – review & editing, Methodology. **Maaike Cima:** Writing – review & editing. **Linn B. Norbom:** Writing – review & editing, Writing – original draft, Methodology. **Yllza Xerxa:** Writing – review & editing, Project administration, Investigation, Data curation. **Sandra Thijssen:** Writing – review & editing, Writing – original draft, Visualization, Methodology, Formal analysis, Conceptualization.

## Declaration of Competing Interest

The authors declare that they have no known competing financial interests or personal relationships that could have appeared to influence the work reported in this paper.

## Data Availability

The data included in the current study are not publicly available due to legal and ethical restrictions. For access to Generation R, researchers can send their request to Vincent Jaddoe (v.jaddoe@erasmusmc.nl).
