# Supplementary material for: Early childhood family threat and longitudinal amygdala-mPFC circuit development: Examining cortical thickness and gray matter-white matter contrast
Source: Dev Cogn Neurosci. 2024 Oct 16;70:101462. doi: 10.1016/j.dcn.2024.101462 (PMC11532282; doi:10.1016/j.dcn.2024.101462)
Supplement: Supplementary file 1 — Supplementary material [file mmc1.docx]

**Supplemental information**

Supplemental Text 1. Deviations from preregistration

The current project was preregistered at: <https://osf.io/yw23h>. On several points, the project has deviated from the original preregistration. First, the preregistration mentions our measure of family conflict will be the average of a prenatal measure and a measure collected at age 5 years. As all other predictors are postnatal, we decided to use only the measure collected at age 5 years. Second, multiple imputation was not performed with MICE in R as preregistered, but was performed in SPSS. Moreover, the preregistration mentions that as a first analysis, developmental trajectories of each outcome will be examined. In this process, an autoregressive error structure will be compared to the default independent error structure. However, to our knowledge, no tools exist to pool effects of linear mixed effect models with autoregressive error structure from multiply imputed datasets. Therefore, although the autoregressive error structure had better fit for rostral ACC cortical thickness, final analyses of this outcome included the default independent error structure. Finally, all analyses mentioned under sensitivity and exploratory analyses in the main manuscript were not preregistered.

Supplemental Text 2. MRI acquisition and preprocessing

Neuroimaging data was collected in a subset of children from the Generation R Study. MRI scanning at the first imaging assessment was performed on a General Electric (GE) Discovery MR 750 3T scanner and has been extensively described in White et al. (2013). A T1-weighted inversion recovery fast spoiled gradient recalled (IR-FSPGR) sequence was obtained using an 8-channel head coil with the following parameters: TR = 10.3 ms, TE = 4.2 ms, TI = 350 ms, NEX = 1, flip angle = 16°, matrix 256x256, imaging acceleration factor 2, and 0.9mm isotropic voxel resolution.

For the second and third assessment, participants were scanned using a different, study-dedicated 3T GE MR750W. A T1-weighted IR-FSPGR sequence was obtained using an 8-channel head coil with the following parameters: TR = 8.77 ms, TE = 3.4 ms, TI = 600 ms, flip angle = 10°, Field of View (FOV) = 220 × 220 mm, acquisition matrix = 220 × 220, slice thickness = 1 mm isotropic, number of slices = 230, ARC acceleration factor = 2 (White et al., 2018). Importantly, as scanner and participant age are correlated, developmental trajectories cannot be meaningfully interpreted. However, with the exception of possible effects of attrition bias, variation in early childhood experiences is not associated with scanner. Therefore, differences in developmental trajectory related to these experiences can be interpreted.

Cortical reconstruction, parcellation and volumetric segmentation was performed using FreeSurfer 6.0 (http://surfer.nmr.mgh.harvard.edu/). Cortical thickness was calculated as the shortest vertex-wise distance between the white surface (i.e. gray/white boundary) and the pial surface (i.e. gray/cerebrospinal fluid boundary) (Fischl & Dale, 2000). GWC was calculated using intensity sampling from the “rawavg.mgz” volume, with white matter sampled 1 mm below-, and grey matter sampled 30% above the white surface. The vertex-wise percentage difference was then computed as 100 × (white − grey)/[(white + grey)/2] so that lower GWC reflects more similar grey and white matter.

Region-of-interest were selected form the Desikan-Killiany Atlas (Desikan et al., 2006). For amygdala volume and GWC measures, mean volume/GWC was created by averaging the left and right hemisphere score. For caudal ACC, rostral ACC and medial OFC thickness, an average thickness measure was created using the following formula: ((left structure thickness * left structure area) + (right structure thickness * right structure area))/(left structure area + right structure area). Data quality assurance consisted of a multistep process including both visual inspection by trained researchers and automated software and is described elsewhere (Muetzel et al., 2019; Steenkamp et al., 2022; Weeland et al., 2021). Only scans of sufficient quality were used for data analysis.

Supplemental Text 2. Confounding variables

Family national origin was defined according to the classification of Statistics Netherlands and categorized as Dutch, non-Dutch European, and non-European. Maternal education level was defined as the highest completed education and was categorized into primary (no or primary education), secondary (lower and intermediate vocational training), and higher (higher vocational education and university) education. Information on family net income was obtained using 10 categories ranging from less than €750 to €3300 or more per month. Maternal psychopathology was measured using the Global Severity Index of the Brief Symptom Inventory when the child was approximately 3 years of age (Derogatis & Melisaratos, 1983). Smoking during pregnancy was categorized as never, until pregnancy was known, occasionally or frequently.

Supplemental Text 4. Exploratory analyses

*Correlations between change in cortical thickness and change in GWC*

Table S1 and Figure S1 show the correlations between change in cortical thickness and change in GWC. Please note that the change from T1-T2 also indicates a change in scanner. There was a negative association between change in caudal ACC cortical thickness and GWC from T2 (+/- 10y) to T3 (+/- 14y), β = -.164. For rostral ACC, there was a positive correlation between change in cortical thickness and GWC, β = .172 and β = .127, for change from T1-T2, and from T2-T3, respectively. For the mOFC, change in cortical thickness and GWC from T2 to T3 was positively correlated, β = .212. The positive correlations suggest that a larger decrease in cortical thickness was associated with a decrease or smaller increase in GWC, whereas smaller decreases or increases in thickness were associated with a smaller decrease or larger increase in GWC.

Table S1. Associations between change in cortical thickness and change in GWC.

|  | T2-T1 (N = 340) | | T3-T2 (N=575) | |
| --- | --- | --- | --- | --- |
|  | β | p | β | p |
| Caudal ACC | -.034 | .462 | -.164 | <.001 |
| Rostral ACC | .172 | <.001 | .127 | <.001 |
| OFC | -.017 | .700 | .212 | <.001 |

Note. Correlations are corrected for age, Δage, and sex; ACC = anterior cingulate cortex; OFC = orbitofrontal cortex


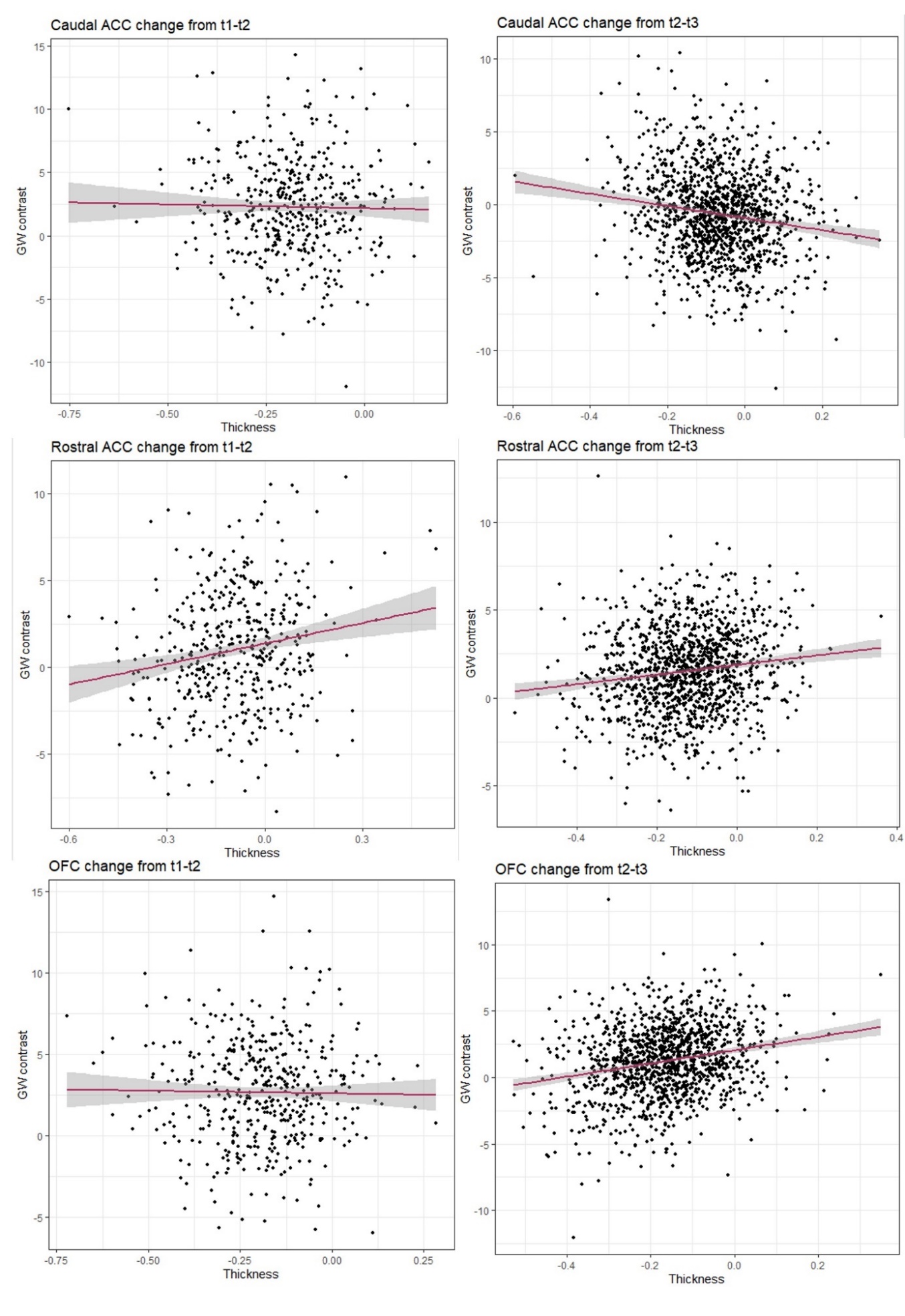
Figure S5. Associations between change in cortical thickness and change in gray-white contrast

Table S2. Mean differences in family factors between wave 1 and subsequent waves

|  | Wave 1 *M* (SD) | Wave 2 *M* (SD) | Wave 3 *M* (SD) |
| --- | --- | --- | --- |
| Harsh parenting | 2.39 (1.96) | 2.12 (1.87)* | 2.12 (1.83)* |
| Family conflict | 1.56 (0.42) | 1.51 (0.42)* | 1.52 (0.43) |
| Neighborhood safety | 7.72 (1.55) | 7.75 (1.59) | 7.71 (1.61) |

Note. * p <.05

Table S3. Correlations between variables (χ^2^ for categorical variables)

|  |  | Family conflict | Safety | Sex | Age t1 | Age t2 | Age t3 | Maternal educaction | Income | Gestational age | Maternal smoking | Maternal psychopahtology |
| --- | --- | --- | --- | --- | --- | --- | --- | --- | --- | --- | --- | --- |
| Harsh parenting | *r* | .153 | -.080 | -.047 | .007 | .012 | .006 | -.145 | -.150 | -.028 | .015 | .264 |
|  | *p* | <.001 | <.001 | 013 | .873 | .580 | .819 | <.001 | <.001 | .144 | .443 | <.001 |
|  | n | 2548 | 2163 | 2759 | 599 | 2079 | 1435 | 2747 | 2456 | 2751 | 2453 | 2736 |
| Family conflict | *r* |  | -.162 | -.038 | .027 | -.012 | .047 | -.200 | -.274 | -.044 | .053 | .253 |
|  | *p* |  | <.001 | .022 | .436 | .544 | .041 | <.001 | <.001 | .009 | .003 | <.001 |
|  | n |  | 2788 | 3560 | 812 | 2586 | 1884 | 3541 | 3359 | 3537 | 3158 | 2576 |
| Safety | *r* |  |  | -.024 | .066 | -.030 | -.061 | .277 | .396 | .034 | -.077 | -.129 |
|  | *p* |  |  | .160 | .082 | .141 | .011 | <.001 | <.001 | .052 | <.001 | <.001 |
|  | n |  |  | 3335 | 699 | 2423 | 1714 | 3222 | 2694 | 3309 | 2898 | 2190 |
| Sex | *r* |  |  |  | -.051 | .062 | .009 | 1.09 | .003 | -.043 | 2.30 | -.018 |
|  | *p* |  |  |  | .125 | <.001 | .682 | .579 | .871 | .005 | .316 | .335 |
|  | n |  |  |  | 901 | 3013 | 2199 | 4079 | 3436 | 4169 | 3701 | 2792 |
| Age t1 | *r* |  |  |  |  | .326 | .359 | .115 | .132 | -.014 | -.189 | -.043 |
|  | *p* |  |  |  |  | <.001 | <.001 | <.001 | <.001 | .683 | <.001 | .290 |
|  | n |  |  |  |  | 455 | 402 | 893 | 795 | 899 | 863 | 606 |
| Age t2 | *r* |  |  |  |  |  | .430 | -.051 | -.035 | -.034 | .041 | .024 |
|  | *p* |  |  |  |  |  | <.001 | .006 | .078 | .060 | .034 | .276 |
|  | n |  |  |  |  |  | 1274 | 2929 | 2512 | 2992 | 2628 | 2109 |
| Age t3 | *r* |  |  |  |  |  |  | -.044 | -.033 | -.060 | -.016 | .006 |
|  | *p* |  |  |  |  |  |  | .041 | .161 | .005 | .485 | .828 |
|  | n |  |  |  |  |  |  | 2133 | 1808 | 2181 | 1942 | 1451 |
| Maternal education | *r* |  |  |  |  |  |  |  | .527 | .060 | 141.93 | -.131 |
|  | *p* |  |  |  |  |  |  |  | <.001 | <.001 | <.001 | <.001 |
|  | n |  |  |  |  |  |  |  | 3418 | 4057 | 3642 | 2778 |
| Family income | *r* |  |  |  |  |  |  |  |  | .058 | -.139 | -.213 |
|  | *p* |  |  |  |  |  |  |  |  | <.001 | <.001 | <.001 |
|  | n |  |  |  |  |  |  |  |  | 3413 | 3046 | 2483 |
| Gestational age | *r* |  |  |  |  |  |  |  |  |  | -.032 | -.019 |
|  | *p* |  |  |  |  |  |  |  |  |  | .052 | .325 |
|  | n |  |  |  |  |  |  |  |  |  | 3701 | 2782 |
| Maternal smoking | *r* |  |  |  |  |  |  |  |  |  |  | .056 |
|  | *p* |  |  |  |  |  |  |  |  |  |  | .005 |
|  | n |  |  |  |  |  |  |  |  |  |  | 2480 |

Note. * *p* < .05; ** *p* < .01*; ** p* <.001

Table S4. Correlations amongst MRI measures

|  |  | Amyg T2 | Amyg T3 | cACC thick T1 | cACC thick T2 | cACC thick T3 | cACC GWC T1 | cACC GWC T2 | cACC GWC T3 | rACC thick T1 | rACC thick T2 | rACC thick T3 | rACC GWC T1 | rACC GWC T2 | rACC GWC T3 | OFC thick T1 | OFC thick T2 | OFC thick T3 | OFC GWC T1 | OFC GWC T2 | OFC GWC T3 |
| --- | --- | --- | --- | --- | --- | --- | --- | --- | --- | --- | --- | --- | --- | --- | --- | --- | --- | --- | --- | --- | --- |
| Amygdala volume T1 | *r* | .807 | .756 | -.115 | -.100 | -.081 | .168 | .033 | .152 | -.230 | -.181 | -.133 | -.002 | -.048 | -.007 | -.199 | -.031 | .102 | .199 | .095 | .102 |
|  | *p* | <.001 | <.001 | <.001 | .033 | .104 | <.001 | .489 | .002 | <.001 | <.001 | .007 | .963 | .310 | .888 | <.001 | .506 | .042 | <.001 | .042 | .041 |
| Amygdala volume T2 | *r* |  | .879 | -.080 | -.081 | -.029 | .072 | .027 | .029 | -.126 | -.148 | -.100 | .004 | -.003 | .026 | -.102 | .003 | .026 | .145 | .204 | .172 |
|  | *p* |  | <.001 | .089 | <.001 | .307 | .124 | .135 | .300 | .007 | <.001 | <.001 | .934 | .890 | .345 | .030 | .882 | .354 | .002 | <.001 | <.001 |
| Amygdala volume T3 | *r* |  |  | -.100 | -.052 | -.025 | .080 | .028 | .038 | -.137 | -.116 | -.074 | .025 | .027 | .023 | -.108 | .043 | .078 | .167 | .198 | .186 |
|  | *p* |  |  | .045 | .064 | .235 | .110 | .315 | .074 | .006 | <.001 | <.001 | .616 | .342 | .275 | .030 | .122 | <.001 | <.001 | <.001 | <.001 |
| Caudal ACC thickness T1 | *r* |  |  |  | .790 | .759 | -.067 | .113 | .159 | .425 | .318 | .317 | -.044 | .018 | .076 | .283 | .118 | .048 | -.115 | -.092 | -.089 |
|  | *p* |  |  |  | <.001 | <.001 | .043 | .016 | .001 | <.001 | <.001 | <.001 | .190 | .709 | .129 | <.001 | .011 | .339 | <.001 | .049 | .074 |
| Caudal ACC thickness T2 | *r* |  |  |  |  | .826 | -.083 | .031 | .091 | .359 | .377 | .366 | -.045 | .010 | .025 | .193 | .198 | .183 | -.089 | -.021 | -.032 |
|  | *p* |  |  |  |  | <.001 | .078 | .091 | .001 | <.001 | <.001 | <.001 | .341 | .578 | .372 | <.001 | <.001 | <.001 | .058 | .258 | .252 |
| Caudal ACC thickness T3 | *r* |  |  |  |  |  | -.067 | .136 | .075 | .321 | .354 | .393 | -.090 | .064 | .012 | .110 | .182 | .208 | -.129 | -.004 | -.023 |
|  | *p* |  |  |  |  |  | .177 | <.001 | <.001 | <.001 | <.001 | <.001 | .072 | .023 | .589 | .028 | <.001 | <.001 | .010 | .879 | .277 |
| Caudal ACC GWC T1 | *r* |  |  |  |  |  |  | .223 | .313 | -.041 | -.001 | .033 | .686 | .092 | .146 | -.158 | .069 | .060 | .631 | .075 | .103 |
|  | *p* |  |  |  |  |  |  | <.001 | <.001 | .220 | .983 | .514 | <.001 | .050 | .003 | <.001 | .141 | .233 | <.001 | .109 | .038 |
| Caudal ACC GWC T2 | *r* |  |  |  |  |  |  |  | .406 | .018 | .059 | .070 | .114 | .549 | .202 | .019 | .069 | .059 | .088 | .441 | .145 |
|  | *p* |  |  |  |  |  |  |  | <.001 | .707 | .001 | .012 | .015 | <.001 | <.001 | .680 | <.001 | .034 | .061 | <.001 | <.001 |
| Caudal ACC GWC T3 | *r* |  |  |  |  |  |  |  |  | .038 | .082 | .116 | .173 | .207 | .480 | -.060 | .046 | .108 | .093 | .102 | .373 |
|  | *p* |  |  |  |  |  |  |  |  | .452 | .003 | <.001 | <.001 | <.001 | <.001 | .233 | .101 | <.001 | .061 | <.001 | <.001 |
| Rostral ACC thickness T1 | *r* |  |  |  |  |  |  |  |  |  | .608 | .558 | .083 | .029 | .108 | .477 | .185 | .134 | -.145 | -.118 | -.056 |
|  | *p* |  |  |  |  |  |  |  |  |  | <.001 | <.001 | .013 | .532 | .031 | <.001 | <.001 | .007 | <.001 | .012 | .259 |
| Rostral ACC thickness T2 | *r* |  |  |  |  |  |  |  |  |  |  | .724 | .027 | .209 | .142 | .278 | .402 | .287 | -.089 | -.046 | .005 |
|  | *p* |  |  |  |  |  |  |  |  |  |  | <.001 | .572 | <.001 | <.001 | <.001 | <.001 | <.001 | .059 | .011 | .845 |
| Rostral ACC thickness T3 | *r* |  |  |  |  |  |  |  |  |  |  |  | .017 | .143 | .185 | .198 | .280 | .431 | -.058 | -.042 | .036 |
|  | *p* |  |  |  |  |  |  |  |  |  |  |  | .736 | <.001 | <.001 | <.001 | <.001 | <.001 | .247 | .138 | .095 |
| Rostral ACC GWC T1 | *r* |  |  |  |  |  |  |  |  |  |  |  |  | .209 | .331 | -.025 | .070 | .041 | .711 | .120 | .172 |
|  | *p* |  |  |  |  |  |  |  |  |  |  |  |  | <.001 | <.001 | .462 | .138 | .407 | <.001 | .010 | <.001 |
| Rostral ACC GWC T2 | *r* |  |  |  |  |  |  |  |  |  |  |  |  |  | .442 | .024 | .015 | .053 | .140 | .622 | .239 |
|  | *p* |  |  |  |  |  |  |  |  |  |  |  |  |  | <.001 | .606 | .410 | .059 | .003 | <.001 | <.001 |
| Rostral ACC GWC T3 |  |  |  |  |  |  |  |  |  |  |  |  |  |  |  | .034 | -.004 | -.015 | .215 | .257 | .606 |
|  |  |  |  |  |  |  |  |  |  |  |  |  |  |  |  | .489 | .875 | .477 | <.001 | <.001 | <.001 |
| OFC thickness T1 | *r* |  |  |  |  |  |  |  |  |  |  |  |  |  |  |  | .456 | .308 | -.105 | .084 | .069 |
|  | *p* |  |  |  |  |  |  |  |  |  |  |  |  |  |  |  | <.001 | <.001 | .002 | .075 | .169 |
| OFC thickness T2 | *r* |  |  |  |  |  |  |  |  |  |  |  |  |  |  |  |  | .575 | .020 | .126 | .087 |
|  | *p* |  |  |  |  |  |  |  |  |  |  |  |  |  |  |  |  | <.001 | .676 | <.001 | .002 |
| OFC thickness T3 | *r* |  |  |  |  |  |  |  |  |  |  |  |  |  |  |  |  |  | .037 | .100 | .260 |
|  | *p* |  |  |  |  |  |  |  |  |  |  |  |  |  |  |  |  |  | .463 | <.001 | <.001 |
| OFC GWC T1 | *r* |  |  |  |  |  |  |  |  |  |  |  |  |  |  |  |  |  |  | .235 | .235 |
|  | *p* |  |  |  |  |  |  |  |  |  |  |  |  |  |  |  |  |  |  | <.001 | <.001 |
| OFC GWC T2 | *r* |  |  |  |  |  |  |  |  |  |  |  |  |  |  |  |  |  |  |  | .371 |
|  | *p* |  |  |  |  |  |  |  |  |  |  |  |  |  |  |  |  |  |  |  | <.001 |

Note. * *p* < .05; ** *p* < .01*; ** p* <.001. n t1-t1 = 901, n t1-t2 = 455, n t1-t3 = 402, n t2-t2 = 3013, n t2-t3 = 1274, n t3-t3 = 2200.

**
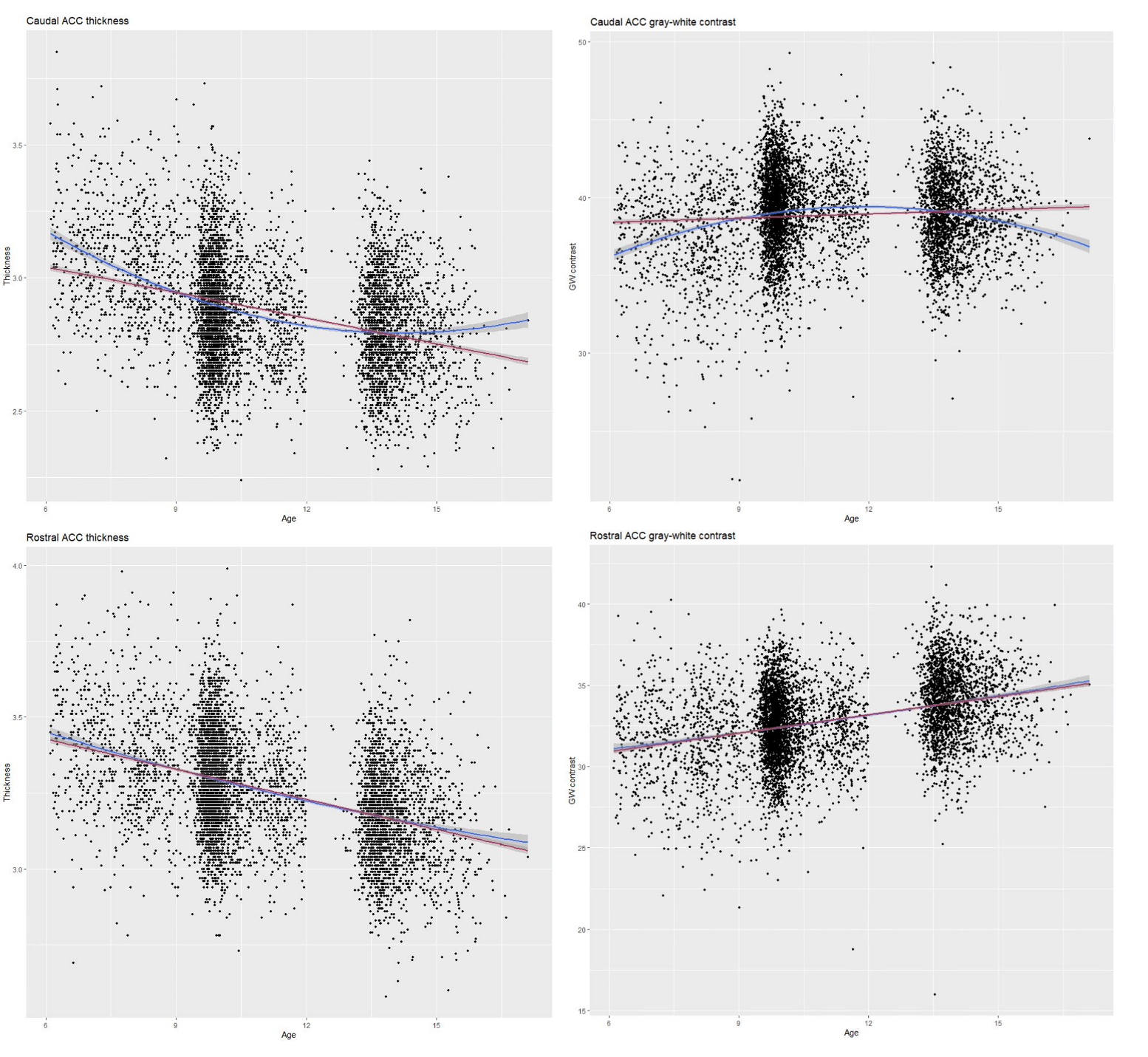
**

**
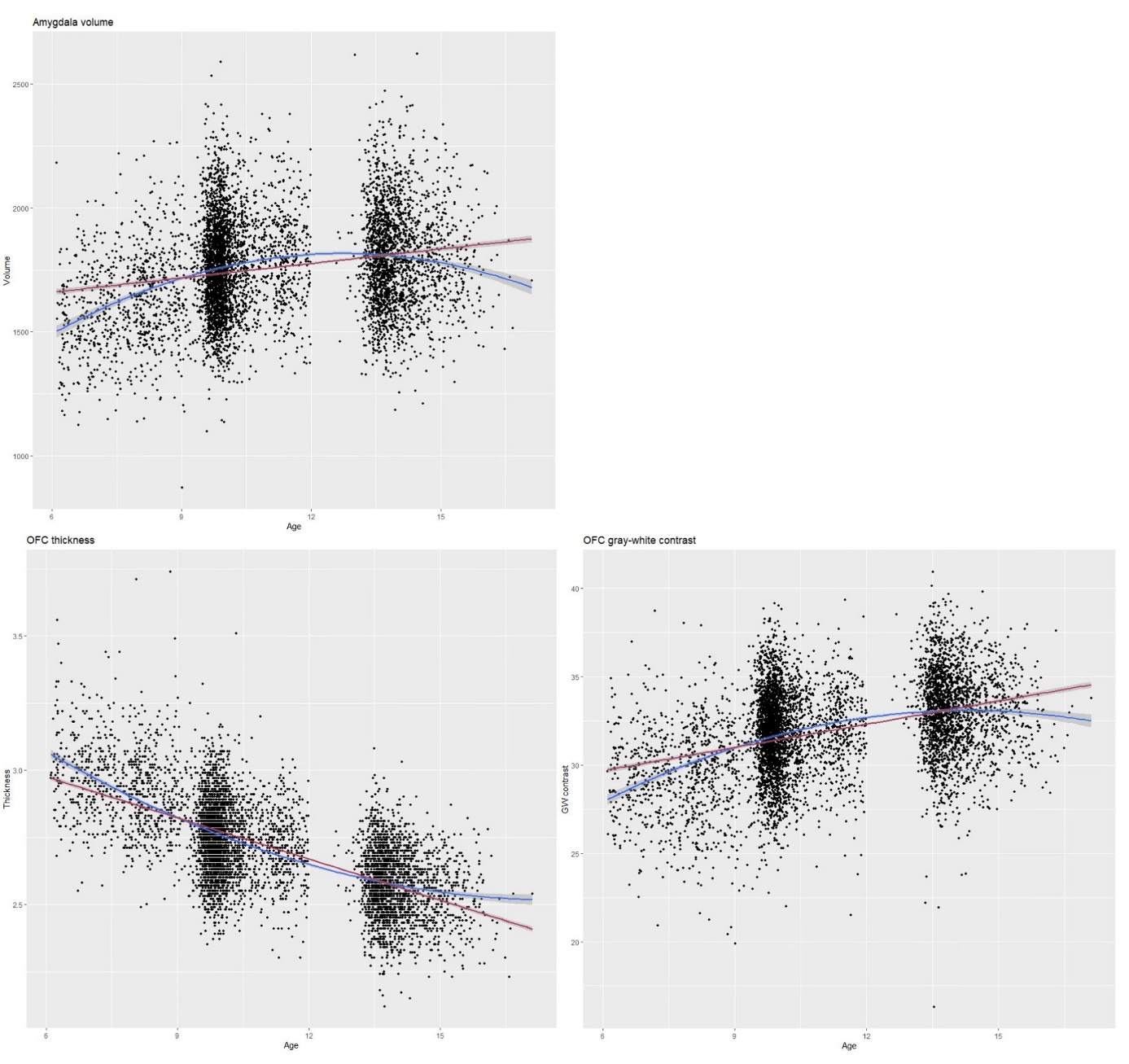
**

Figure S2. Developmental trajectories of amygdala-mPFC circuit structure. Linear association in red, quadratic association in blue.

Table S5. Model selection criteria: age vs age^2^

|  | Age | | Age^2^ | |
| --- | --- | --- | --- | --- |
|  | AIC | LL | AIC | LL (p) |
| Amygdala | 80013.54 | -40003 | **79486.12** | **-39738 (<.001)** |
| Caudal ACC thickness | -4028.27 | 2018.1 | **-4330.78** | **2170.4 (<.001)** |
| Caudal ACC gwc | 30219.25 | -15106 | **30025.61** | **-15008 (<.001)** |
| Rostral ACC thickness | **-5758.03** | **2883.0** | -5744.29 | 2877.1 (<.001) |
| Rostral ACC gwc | **27727.31** | **-13860** | 27735.48 | -13863 (.013) |
| OFC thickness | -7462.77 | 3735.4 | **-7624.05** | **3817.0 (<.001)** |
| OFC gwc | 28293.17 | -14143 | **28132.52** | **-14061 (<.001)** |

Note. AIC = Akaike Information Criterion; LL = Log-likelihood

Table S6. Partial correlations between family factors and brain structure.

| Family factor | Structure | T1 (5-10y) | T2 (10y) | T3 (13y) |
| --- | --- | --- | --- | --- |
|  |  | *r* | *r* | *r* |
| Harsh parenting | Amygdala volume | -.067 | -.060 | -.061 |
|  | Caudal ACC thickness | .036 | -.015 | .001 |
|  | Caudal ACC GWC | .010 | .030 | -.030 |
|  | Rostral ACC thickness | -.015 | .001 | -.064 |
|  | Rostral ACC GWC | -.040 | .035 | -.039 |
|  | OFC thickness | -.050 | -.008 | -.019 |
|  | OFC GWC | -.026 | .010 | -.049 |
| Family conflict | Amygdala volume | -.019 | .013 | -.011 |
|  | Caudal ACC thickness | .021 | .002 | -.050 |
|  | Caudal ACC GWC | .012 | -.007 | .015 |
|  | Rostral ACC thickness | -.022 | .028 | .012 |
|  | Rostral ACC GWC | -.040 | .034 | .022 |
|  | OFC thickness | -.014 | .043 | -.004 |
|  | OFC GWC | -.026 | .010 | -.040 |
| Neighborhood safety | Amygdala volume | .033 | .060 | .040 |
|  | Caudal ACC thickness | -.106 | .016 | -.007 |
|  | Caudal ACC GWC | -.002 | -.022 | .000 |
|  | Rostral ACC thickness | -.027 | .014 | .027 |
|  | Rostral ACC GWC | .001 | -.026 | .026 |
|  | OFC thickness | -.024 | -.057 | -.040 |
|  | OFC GWC | .053 | -.028 | -.007 |

Note. Correlations are corrected for age, sex, ethnicity, maternal education, family income, maternal psychopathology, prenatal smoking, gestational age at birth. ACC = anterior cingulate cortex; GWC = gray-white contrast; OFC = orbitofrontal cortex

Table S7. Linear mixed effect models of harsh parenting – imaging assessment 2 and 3 only

| Structure | Effect | Model 1 | | Model 2 | | Model 3 | |
| --- | --- | --- | --- | --- | --- | --- | --- |
|  |  | β | p | β | p | β | p |
| Amygdala volume (TBV corr) | Age interaction | 0.03 | .196 | 0.03 | .193 | 0.02 | .476 |
|  | Main effect | -0.02 | .242 | -0.03 | .132 | -0.04 | .077 |
| Amygdala volume | Age interaction | 0.01 | .490 | 0.01 | .488 | -0.00 | .955 |
|  | Main effect | -0.04 | .012 | -0.05 | .008 | -0.06 | .008 |
| Caudal ACC thickness | Age interaction | 0.01 | .501 | 0.01 | .490 | 0.03 | .309 |
|  | Main effect | -0.02 | .203 | -0.03 | .128 | -0.01 | .747 |
| Caudal ACC GWC | Age interaction | -0.01 | .541 | -0.01 | .567 | -0.02 | .378 |
|  | Main effect | 0.01 | .610 | 0.01 | .543 | 0.01 | .708 |
| Rostral ACC thickness | Age interaction | -0.01 | .231 | -0.01 | .242 | -0.02 | .183 |
|  | Main effect | -0.02 | .211 | -0.03 | .162 | -0.02 | .314 |
| Rostral ACC GWC | Age interaction | -0.02 | .305 | -0.02 | .339 | -0.03 | .174 |
|  | Main effect | 0.01 | .611 | -0.01 | .556 | 0.00 | .875 |
| mOFC thickness | Age interaction | -0.02 | .113 | -0.02 | .116 | -0.01 | .293 |
|  | Main effect | -0.01 | .389 | -0.01 | .362 | -0.01 | .441 |
| mOFC GWC | Age interaction | -0.01 | .642 | -0.01 | .656 | -0.02 | .328 |
|  | Main effect | -0.02 | .152 | -0.02 | .222 | -0.01 | .513 |

Note: model 1: adjusted for sex, family national origin, family income, and maternal education.
Model 2 = model 1 + gestational age, maternal psychopathology, and maternal smoking during pregnancy.
Model 3 = model 2 + family conflict, neighborhood safety
ACC = anterior cingulate cortex; GWC = gray-white contrast; mOFC = medial orbitofrontal cortex


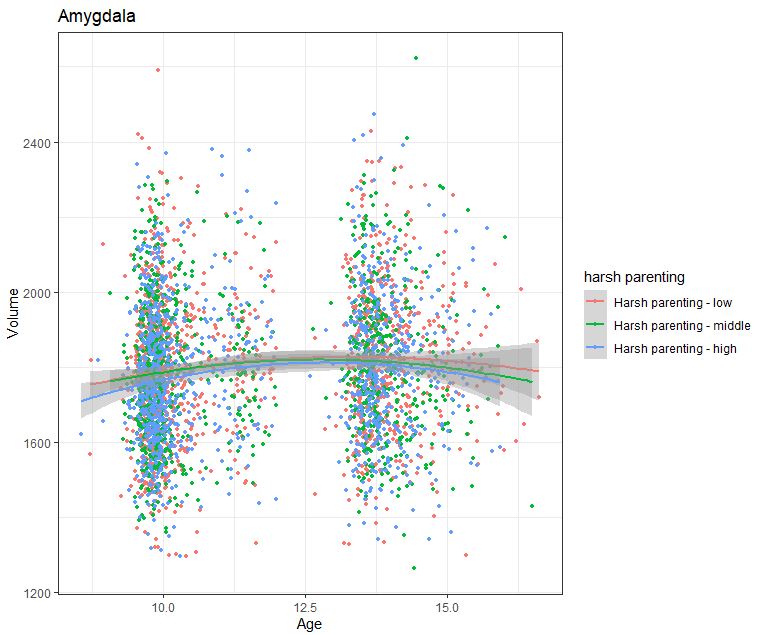


Figure S3. Association between harsh parenting and amygdala volume – imaging assessment 2 and 3 only

Table S8. Linear mixed effect models of family conflict – imaging assessment 2 and 3 only

| Structure | Effect | Model 1 | | Model 2 | | Model 3 | |
| --- | --- | --- | --- | --- | --- | --- | --- |
|  |  | β | p | β | p | β | p |
| Amygdala volume (TBV corr) | Age interaction | -0.03 | .087 | -0.03 | .089 | -0.04 | .240 |
|  | Main effect | 0.01 | .437 | 0.01 | .612 | 0.03 | .277 |
| Amygdala volume | Age interaction | -0.02 | .136 | -0.02 | .146 | -0.03 | .277 |
|  | Main effect | -0.00 | .778 | -0.01 | .668 | 0.01 | .709 |
| Caudal ACC thickness | Age interaction | -0.02 | .020 | -0.02 | .026 | -0.02 | .183 |
| Caudal ACC GWC | Age interaction | 0.03 | .032 | 0.03 | .033 | 0.02 | .320 |
| Rostral ACC thickness | Age interaction | 0.01 | .376 | 0.01 | .372 | -0.00 | .968 |
|  | Main effect | 0.00 | .937 | 0.00 | .979 | 0.03 | .217 |
| Rostral ACC GWC | Age interaction | -0.01 | .508 | -0.01 | .509 | -0.01 | .453 |
|  | Main effect | 0.00 | .938 | 0.00 | .919 | 0.03 | .158 |
| OFC thickness | Age interaction | 0.01 | .364 | 0.01 | .376 | -0.01 | .340 |
|  | Main effect | 0.01 | .524 | 0.01 | .515 | 0.02 | .258 |
| OFC GWC | Age interaction | -0.00 | .788 | -0.01 | .710 | -0.03 | .152 |
|  | Main effect | -0.01 | .740 | -0.01 | .703 | -0.01 | .770 |

Note: model 1: adjusted for sex, family national origin, family income, and maternal education.
Model 2 = model 1 + gestational age, maternal psychopathology, and maternal smoking during pregnancy.
Model 3 = model 2 + harsh parenting, neighborhood safety
ACC = anterior cingulate cortex; GWC = gray-white contrast; OFC = orbitofrontal cortex


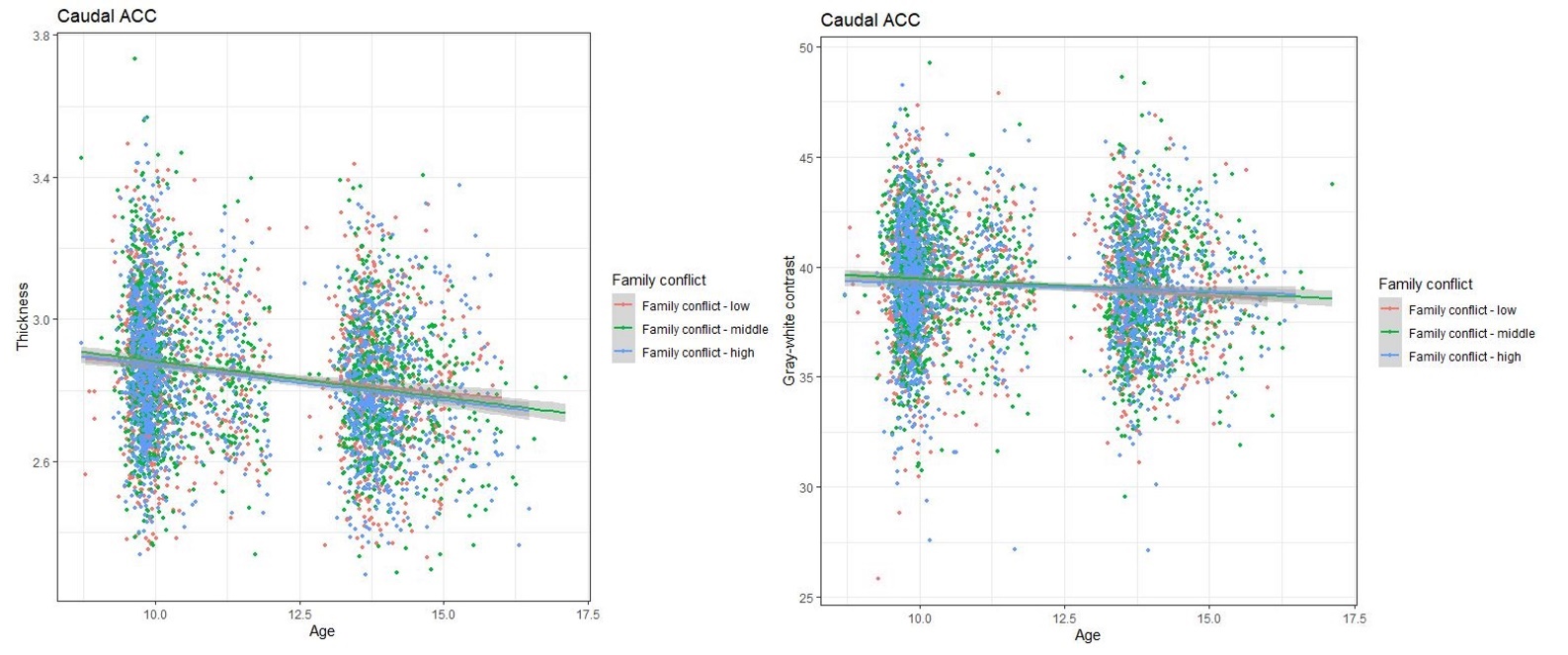


Figure S4. Associations between family conflict and caudal ACC development – imaging assessment 2 and 3 only

Table S9. Linear mixed effect models of neighborhood safety – imaging assessment 2 and 3 only

| Structure | Effect | Model 1 | | Model 2 | | Model 3 | |
| --- | --- | --- | --- | --- | --- | --- | --- |
|  |  | β | p | β | p | β | p |
| Amygdala volume (TBV corr) | Age interaction | -0.02 | .462 | -0.02 | .449 | -0.02 | .569 |
|  | Main effect | 0.05 | .015 | 0.05 | .012 | 0.06 | .017 |
| Amygdala volume | Age interaction | -0.01 | .550 | -0.01 | .547 | -0.01 | .672 |
|  | Main effect | 0.05 | .002 | 0.05 | .003 | 0.05 | .020 |
| Caudal ACC thickness | Age interaction | 0.01 | .340 | 0.01 | .364 | -0.01 | .519 |
|  | Age^2^ interaction | -0.04 | .037 | -0.04 | .041 | -0.03 | .191 |
|  | Main effect | 0.01 | .775 | 0.01 | .695 | -0.00 | .973 |
| Caudal ACC GWC | Age interaction | 0.01 | .554 | 0.01 | .541 | 0.01 | .457 |
|  | Age^2^ interaction | -0.04 | .117 | -0.04 | .116 | -0.07 | .031 |
|  | Main effect | -0.01 | .546 | -0.01 | .574 | -0.01 | .582 |
| Rostral ACC thickness | Age interaction | 0.01 | .191 | 0.01 | .202 | 0.02 | .231 |
|  | Main effect | -0.00 | .911 | -0.00 | .950 | 0.01 | .691 |
| Rostral ACC GWC | Age interaction | 0.04 | .006 | 0.04 | .007 | 0.04 | .040 |
| mOFC thickness | Age interaction | -0.00 | .742 | -0.00 | .748 | -0.01 | .442 |
|  | Main effect | -0.03 | .035 | -0.03 | .035 | -0.04 | .024 |
| mOFC GWC | Age interaction | 0.01 | .363 | 0.01 | .378 | 0.02 | .358 |
|  | Age^2^ interaction | -0.04 | .089 | -0.04 | .094 | -0.09 | .009 |
|  | Main effect | -0.02 | .340 | -0.02 | .340 | -0.02 | .362 |

Note: model 1: adjusted for sex, family national origin, family income, and maternal education.
Model 2 = model 1 + gestational age, maternal psychopathology, and maternal smoking during pregnancy.
Model 3 = model 2 + harsh parenting, family conflict
ACC = anterior cingulate cortex; GWC = gray-white contrast; OFC = orbitofrontal cortex


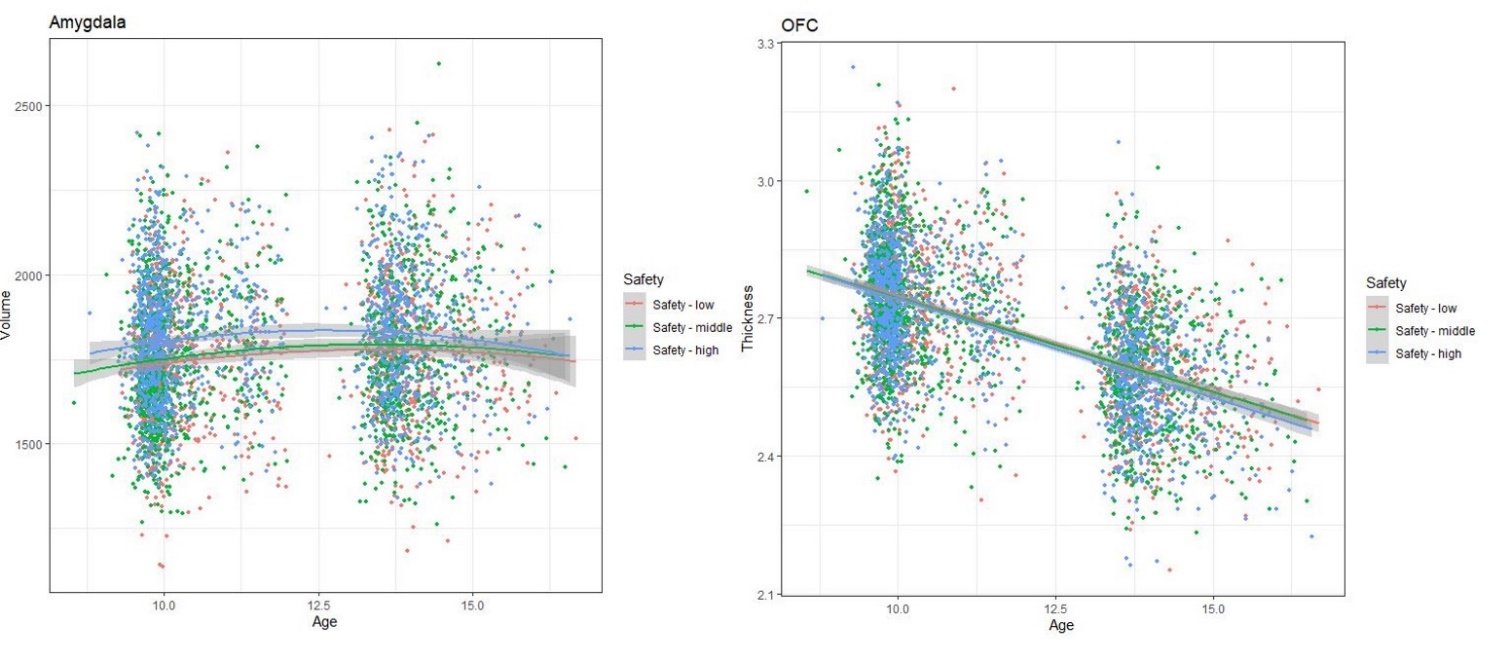


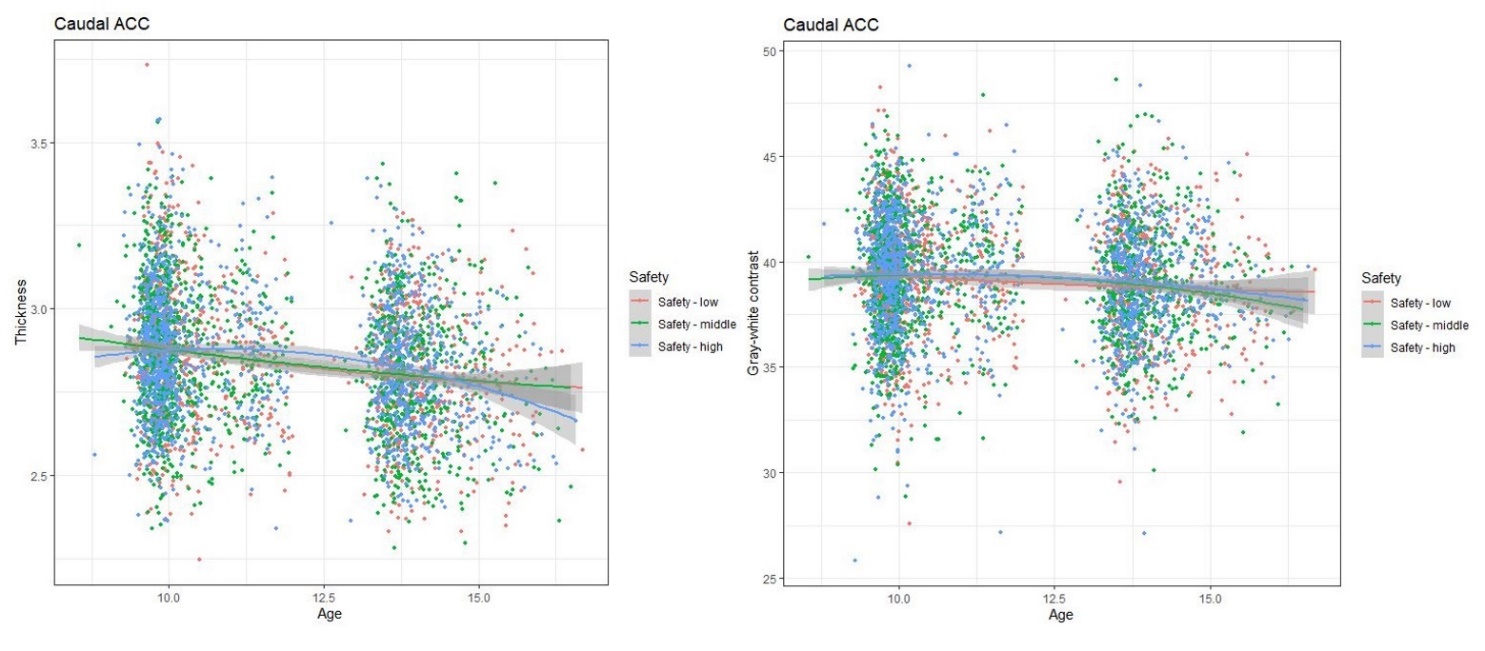


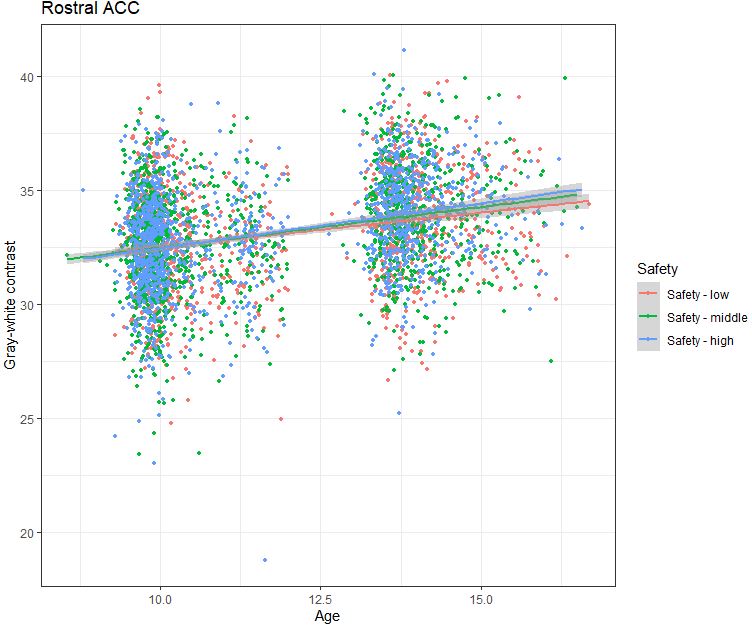


Figure S5. Associations between safety and amygdala, OFC and ACC development – imaging assessment 2 and 3 only

Table S10. Linear mixed effect models of harsh parenting – correction for surface hole number

| Structure | Effect | Model 1 | | Model 2 | | Model 3 | |
| --- | --- | --- | --- | --- | --- | --- | --- |
|  |  | β | p | β | p | β | p |
| Amygdala volume (TBV corr) | Age interaction | 0.00 | .688 | 0.00 | .692 | -0.01 | .688 |
|  | Main effect | -0.03 | .077 | -0.04 | .045  (.158) | -0.04 | .060 |
| Amygdala volume | Age interaction | 0.00 | .613 | 0.00 | .637 | -0.00 | .657 |
|  | Main effect | -0.06 | <.001  (.001) | -0.06 | <.001  (.001) | -0.06 | .003  (.021) |
| Caudal ACC thickness | Age interaction | 0.00 | .828 | 0.00 | .834 | 0.00 | .997 |
|  | Main effect | -0.00 | .863 | -0.01 | .524 | 0.01 | .807 |
| Caudal ACC GWC | Age interaction | 0.01 | .305 | 0.02 | .296 | 0.03 | .093 |
|  | Main effect | -0.01 | .595 | -0.01 | .776 | -0.01 | .816 |
| Rostral ACC thickness | Age interaction | -0.01 | .467 | -0.01 | .490 | -0.01 | .581 |
|  | Main effect | -0.01 | .476 | -0.02 | .356 | -0.02 | .332 |
| Rostral ACC GWC | Age interaction | -0.02 | .250 | -0.02 | .259 | -0.02 | .174 |
|  | Main effect | 0.00 | .829 | 0.00 | .949 | -0.01 | .629 |
| mOFC thickness | Age interaction | -0.01 | .389 | -0.01 | .402 | -0.01 | .659 |
|  | Main effect | -0.01 | .723 | -0.01 | .698 | -0.01 | .524 |
| mOFC GWC | Age interaction | 0.00 | .958 | 0.00 | .967 | -0.01 | .759 |
|  | Main effect | -0.04 | .003  (.010) | -0.04 | .011  (.039) | -0.03 | .079 |

Note: model 1: adjusted for sex, family national origin, family income, and maternal education.
Model 2 = model 1 + gestational age, maternal psychopathology, and maternal smoking during pregnancy.
Model 3 = model 2 + family conflict, neighborhood safety
ACC = anterior cingulate cortex; GWC = gray-white contrast; OFC = orbitofrontal cortex

Table S11. Linear mixed effect models of family conflict - correction for surface hole number

| Structure | Effect | Model 1 | | Model 2 | | Model 3 | |
| --- | --- | --- | --- | --- | --- | --- | --- |
|  |  | β | p | β | p | β | p |
| Amygdala volume (TBV corr) | Age interaction | -0.01 | .222 | -0.01 | .226 | -0.02 | .147 |
|  | Main effect | 0.01 | .632 | 0.01 | .785 | 0.02 | .297 |
| Amygdala volume | Age interaction | -0.01 | .439 | -0.01 | .449 | -0.01 | .266 |
|  | Main effect | -0.01 | .540 | -0.01 | .518 | 0.01 | .640 |
| Caudal ACC thickness | Age interaction | -0.02 | .012  (.042) | -0.02 | .015  (.052) | -0.02 | .096 |
| Caudal ACC GWC | Age interaction | 0.04 | .004  (.028) | 0.04 | .004  (.028) | 0.03 | .046  (.322) |
| Rostral ACC thickness | Age interaction | 0.00 | .844 | 0.00 | .784 | -0.00 | .837 |
|  | Main effect | 0.01 | .625 | 0.01 | .754 | 0.02 | .276 |
| Rostral ACC GWC | Age interaction | -0.01 | .589 | -0.01 | .586 | -0.00 | .887 |
|  | Main effect | -0.00 | .865 | -0.00 | .890 | 0.02 | .418 |
| mOFC thickness | Age interaction | -0.01 | .614 | -0.01 | .607 | -0.00 | .781 |
|  | Main effect | 0.01 | .305 | 0.01 | .324 | 0.02 | .331 |
| mOFC GWC | Age interaction | 0.01 | .264 | 0.01 | .278 | -0.00 | .976 |
|  | Main effect | -0.01 | .349 | -0.01 | .397 | -0.01 | .465 |

Note: model 1: adjusted for sex, family national origin, family income, and maternal education.
Model 2 = model 1 + gestational age, maternal psychopathology, and maternal smoking during pregnancy.
Model 3 = model 2 + harsh parenting, neighborhood safety
ACC = anterior cingulate cortex; GWC = gray-white contrast; OFC = orbitofrontal cortex

| Structure | Effect | Model 1 | | Model 2 | | Model 3 | |
| --- | --- | --- | --- | --- | --- | --- | --- |
|  |  | Β (SE) | p | β | p | β | p |
| Amygdala volume (TBV corr) | Age interaction | -0.00 | .846 | -0.00 | .868 | 0.01 | .292 |
|  | Main effect | 0.04 | .022 (.055) | 0.05 | .017  (.043) | 0.06 | .011  (.030) |
| Amygdala volume | Age interaction | -0.00 | .739 | 0.00 | .759 | 0.01 | .355 |
|  | Main effect | 0.06 | .001  (.005) | 0.06 | .001  (.005) | 0.06 | .008  (.030) |
| Caudal ACC thickness | Age interaction | 0.00 | .992 | -0.00 | .934 | -0.03 | .012  (.084) |
|  | Main effect | -0.01 | .733 | -0.00 | .817 | -0.01 | .536 |
| Caudal ACC GWC | Age interaction | -0.03 | .032  (.112) | -0.03 | .035  (.123) | -0.02 | .389 |
| Rostral ACC thickness | Age interaction | 0.02 | .058 | 0.02 | .063 | 0.02 | .055 |
|  | Main effect | -0.01 | .483 | -0.01 | .535 | 0.00 | .995 |
| Rostral ACC GWC | Age interaction | 0.05 | <.001  (.001) | 0.05 | <.001  (.001) | 0.04 | .032  (.112) |
| mOFC thickness | Age interaction | -0.01 | .481 | -0.01 | .486 | -0.02 | .158 |
|  | Main effect | -0.04 | .006  (.015) | -0.04 | .007  (.018) | -0.04 | .012  (.030) |
| mOFC GWC | Age interaction | -0.01 | .446 | -0.01 | .459 | 0.00 | .873 |
|  | Main effect | -0.00 | .956 | -0.00 | .947 | -0.00 | .883 |
|  |  |  |  |  |  |  |  |

Table S12. Linear mixed effect models of neighborhood safety - correction for surface hole number

Note: model 1: adjusted for sex, family national origin, family income, and maternal education.
Model 2 = model 1 + gestational age, maternal psychopathology, and maternal smoking during pregnancy.
Model 3 = model 2 + harsh parenting, family conflict
ACC = anterior cingulate cortex; GWC = gray-white contrast; OFC = orbitofrontal cortex

Table S13. Significant associations corrected for thickness/GWC

| Family factors | Structure | Effect | Model 1 | | Model 2 | | Model 3 | |
| --- | --- | --- | --- | --- | --- | --- | --- | --- |
|  |  |  | β | p | β | p | β | p |
| Harsh parenting | OFC GWC | Main effect | -0.04 | .017 | -0.03 | .041 | -0.02 | .256 |
| Family conflict | Caudal ACC thickness | Age interaction | -0.02 | .029 | -0.02 | .035 | -0.02 | .134 |
|  | Caudal ACC GWC | Age interaction | 0.03 | .010 | 0.03 | .011 | 0.03 | .057 |
| Neighborhood safety | Caudal ACC thickness | Age interaction | -0.01 | .241 | 0.01 | .255 | -0.02 | .082 |
|  | Caudal ACC GWC | Age interaction | -0.03 | .064 | -0.03 | .070 | -0.01 | .563 |
|  | Rostral ACC thickness | Age interaction | 0.01 | .306 | 0.01 | .316 | 0.02 | .172 |
|  | Rostral ACC GWC | Age interaction | 0.05 | <.001 | 0.05 | .001 | 0.03 | .055 |
|  | OFC thickness | Main effect | -0.03 | .020 | -0.03 | .021 | -0.04 | .038 |

Note: model 1: adjusted for sex, family national origin, family income, and maternal education.
Model 2 = model 1 + gestational age, maternal psychopathology, and maternal smoking during pregnancy.
Model 3 = model 2 + harsh parenting and/or family conflict and/or neighborhood safety
ACC = anterior cingulate cortex; GWC = gray-white contrast; OFC = orbitofrontal cortex
